# Advances in Light-Responsive Smart Multifunctional Nanofibers: Implications for Targeted Drug Delivery and Cancer Therapy

**DOI:** 10.3390/pharmaceutics16081017

**Published:** 2024-07-31

**Authors:** Ahmed M. Agiba, Nihal Elsayyad, Hala N. ElShagea, Mahmoud A. Metwalli, Amin Orash Mahmoudsalehi, Saeed Beigi-Boroujeni, Omar Lozano, Alan Aguirre-Soto, Jose Luis Arreola-Ramirez, Patricia Segura-Medina, Raghda Rabe Hamed

**Affiliations:** 1 School of Engineering and Sciences, Tecnologico de Monterrey, Monterrey 64849, Mexico; ahmed.agiba@tec.mx (A.M.A.); amsorash@gmail.com (A.O.M.); alan.aguirre@itesm.mx (A.A.-S.); 2 Department of Pharmaceutics and Industrial Pharmacy, Faculty of Pharmacy, October for Modern Sciences and Arts University, Cairo 12451, Egypt; nmahdy@msa.edu.eg; 3 Department of Pharmaceutics and Industrial Pharmacy, Faculty of Pharmacy, Ahram Canadian University, Cairo 12451, Egypt; hala.nehad@acu.edu.eg; 4 El Demerdash Hospital, Faculty of Medicine, Ain Shams University, Cairo 11591, Egypt; mammkbih@gmail.com; 5 School of Medicine and Health Sciences, Tecnológico de Monterrey, Monterrey 64849, Mexico; omar.lozano@tec.mx; 6 Institute for Obesity Research, Tecnológico de Monterrey, Monterrey 64849, Mexico; 7 Department of Bronchial Hyperresponsiveness, National Institute of Respiratory Diseases “Ismael Cosío Villegas”, Mexico City 14080, Mexico; arreolaj2002@yahoo.com.mx; 8 School of Medicine and Health Sciences, Tecnológico de Monterrey, Mexico City 14380, Mexico; 9 Department of Industrial Pharmacy, College of Pharmaceutical Sciences and Drug Manufacturing, Misr University for Science and Technology, Cairo 12566, Egypt; raghda.hamid@must.edu.eg

**Keywords:** nanofiber-based drug delivery systems, electrospinning, forcespinning, light-responsive smart nanofibers, dual-stimuli-responsive smart nanofibers, surface functionalization, nano-in-nanofiber emerging delivery systems, cancer therapy

## Abstract

Over the last decade, scientists have shifted their focus to the development of smart carriers for the delivery of chemotherapeutics in order to overcome the problems associated with traditional chemotherapy, such as poor aqueous solubility and bioavailability, low selectivity and targeting specificity, off-target drug side effects, and damage to surrounding healthy tissues. Nanofiber-based drug delivery systems have recently emerged as a promising drug delivery system in cancer therapy owing to their unique structural and functional properties, including tunable interconnected porosity, a high surface-to-volume ratio associated with high entrapment efficiency and drug loading capacity, and high mass transport properties, which allow for controlled and targeted drug delivery. In addition, they are biocompatible, biodegradable, and capable of surface functionalization, allowing for target-specific delivery and drug release. One of the most common fiber production methods is electrospinning, even though the relatively two-dimensional (2D) tightly packed fiber structures and low production rates have limited its performance. Forcespinning is an alternative spinning technology that generates high-throughput, continuous polymeric nanofibers with 3D structures. Unlike electrospinning, forcespinning generates fibers by centrifugal forces rather than electrostatic forces, resulting in significantly higher fiber production. The functionalization of nanocarriers on nanofibers can result in smart nanofibers with anticancer capabilities that can be activated by external stimuli, such as light. This review addresses current trends and potential applications of light-responsive and dual-stimuli-responsive electro- and forcespun smart nanofibers in cancer therapy, with a particular emphasis on functionalizing nanofiber surfaces and developing nano-in-nanofiber emerging delivery systems for dual-controlled drug release and high-precision tumor targeting. In addition, the progress and prospective diagnostic and therapeutic applications of light-responsive and dual-stimuli-responsive smart nanofibers are discussed in the context of combination cancer therapy.

## 1. General Introduction

Pharmaceutical nanotechnology has emerged as one of the most rapidly growing fields of science and technology, mainly dealing with nanoscale functional materials and nano-delivery systems (10–1000 nm) [1]. With the advancement of pharmaceutical nanotechnology, new avenues for cancer treatment have become possible through the development of various smart materials that were highly effective in overcoming the problems associated with traditional chemotherapy, which experienced deficiencies of poor aqueous solubility and bioavailability, and a lack of selectivity and specificity, resulting in unsatisfactory therapeutic outcomes and serious side effects [2]. The term “smart materials” was first coined by Toshinori Takagi in 1990 and was defined as materials whose physical properties can change in response to external stimuli [3]. At that time, the scope and feasibility of this concept were unclear, but it was expected to open up an unexplored arena in research and development. Nowadays, the term “smart materials” refers to “stimuli-responsive materials”, which have gained popularity among researchers as technology advances and novel materials are required to meet new regulatory standards. With the advancement of research, the usage of stimuli has expanded to include external stimuli such as light, electric fields, magnetic fields, and ultrasound, as well as internal stimuli such as pH, temperature, redox, and enzymes [4]. Among these smart materials are fibrous materials, which have several distinct advantages in terms of payload loading and responsive release and can be customized for specific functionalities [5]. Nanofibers are filamentous or thread-like structures, often at the nanoscale in diameter, composed of natural or synthetic polymers, or a combination of both, designed for controlled drug delivery and targeting. Their similarity to the extracellular matrix (ECM)-like structure in terms of cell adhesion, proliferation, and differentiation makes them a potential candidate for cancer therapy [6]. In addition, due to their high porosity, surface area-to-volume ratio, ease of fabrication and functionalization, and high tunability [5,6], their use as an anticancer drug delivery system has attracted much interest for potential therapeutic and diagnostic applications. However, these systems pose major challenges in terms of fiber quality and production rate, morphology and surface roughness, structural integrity and mechanical strength, surface functionalization, and stability [5,6]. Initial research on the nanofiber structure revealed a smooth surface with a solid core. However, in recent years, a variety of nanofiber topologies have emerged with potential use in drug delivery and cancer therapy. This review exhaustively portrays the latest advances in developing electro- and forcespun smart nanofibers, including light-responsive and dual-stimuli-responsive smart nanofibers. Special attention is dedicated to multifunctional, nano-in-nanofiber emerging delivery systems due to their highly tunable, dual-controlled release properties, and high-precision tumor targeting. 

## 2. Electrospinning and Electrospun Nanofibers

### 2.1. The Concept of Electrospinning

**Figure 1:**
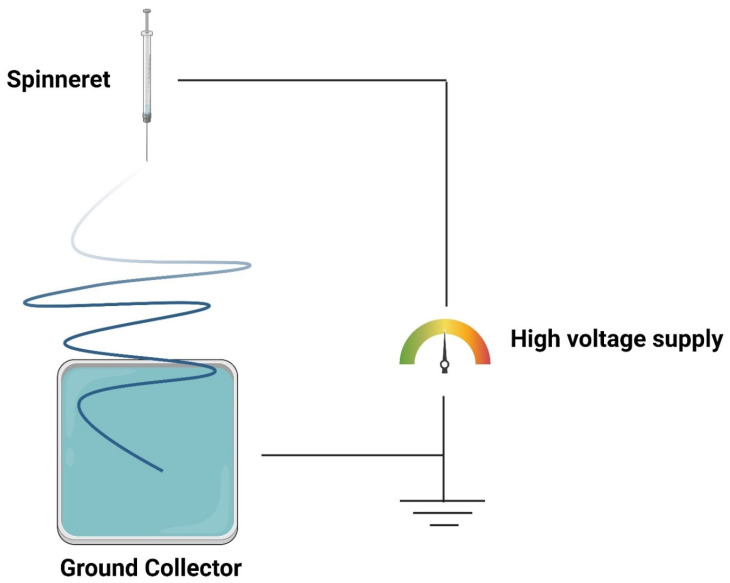
The basic electrospinning setup. Created with BioRender.com.

Electrospinning, first described in 1899 [7], regained popularity in the 1990s, owing primarily to the work of Reneker and Doshi [8], who coined the term “electrospinning” and demonstrated the viability of this method for generating long and continuous fibers with diameters as small as nanometers using various polymers. Electrospinning is a fiber production method that uses electrical force to generate charged threads from a specific polymeric solution or melt. It operates by delivering a high voltage between the surface of the polymeric solution and the collector, causing the solution surface to break down by overcoming its surface tension, resulting in the formation of a thin jet of solution and a cone-like structure known as the “Taylor cone” [1,5,6]. The solvent eventually evaporates, and the resulting solid material is deposited on the collector in the form of nano-sized fibers, as shown in Figure 1.

**Figure 2:**
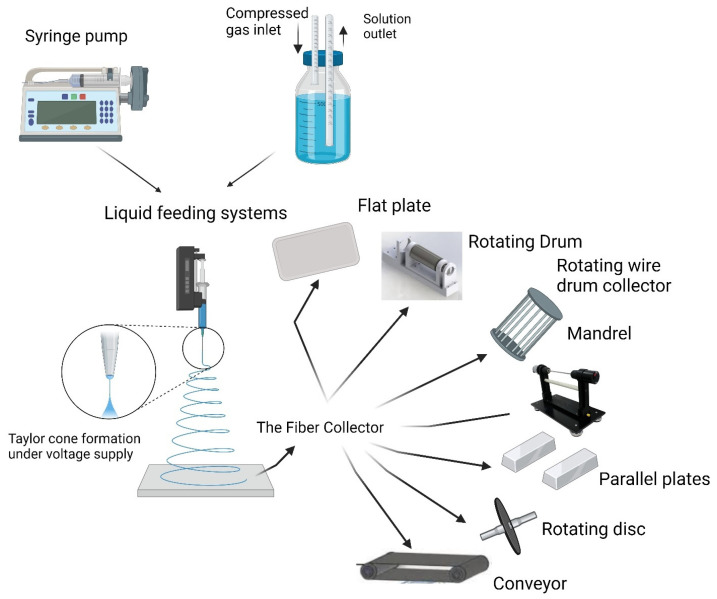
The various methods used for liquid feeding and fiber collection during the electrospinning process. Created with BioRender.com.

Generally, electrospinning requires a conductive collector, a spinneret, a syringe pump, and a high-voltage power supply to operate [9]. Two major systems can be used for feeding polymeric solutions during the electrospinning process. The first is the syringe pump system, which is the most commonly used method to deliver polymeric solutions. It can deliver up to 140 mL of solution per batch with varying flow rates. In this system, solutions are contained in glass or plastic syringes. The other system used is the pressurized liquid feeding system, which can contain multiple volumes of solutions and can be operated in a semi-continuous mode. In that system, solutions are contained in a reservoir, from which they are delivered at a constant flow rate [10,11]. The choice between using the syringe pump or pressurized liquid feeding systems depends on the specific requirements of the electrospinning process, such as the volume of the polymeric solution used and the desired flow rate. On the other hand, there are several types of collectors used in electrospun fiber collection. The proper choice of the collector type is crucial and plays a significant role in the structural and functional design of electrospun nanofibers. Types of collectors that are commonly used in electrospinning include flat plate collectors, drum collectors, mandrel collectors, parallel plate disk collectors, and roll-to-roll collectors [12,13]. The variation in design and functionality between these collectors allows for adjusting the fiber collection process with different orientations and structures to meet any specific requirements in the fabrication process [14,15]. Figure 2 depicts the various methods used for liquid feeding and fiber collection during the electrospinning process. 

To date, electrospun nanofibers have successfully incorporated a wide range of therapeutics, from small-molecule drugs to large biological macromolecules [8,12,15]. However, it is worth noting that all electrospun nanofiber-based systems for cancer research are currently in the preclinical or early stages.

### 2.2. Factors Affecting Electrospun Nanofiber Fabrication

Because of the complexity of the electrospinning concept, a number of parameters should be adjusted to control the diameter, morphology, structure, composition, and alignment of the generated nanofibers. The nanofiber diameter could be adjusted by increasing the voltage difference to decrease it, or by increasing the rate of solution delivery to increase it. The increase in the space between the solution surface and the collector decreases nanofiber diameter, whereas a decrease in the space between the solution surface and the collector or an increase in the rate of polymer solution can result in solvent non-evaporation, increasing the nanofiber diameter, or obtaining bead-like structures [10,14]. The surface tension and electrostatic charge repulsion in the jet result in “Rayleigh instability”, which occurs when two opposing forces exist. The feed solution should be viscous enough to withstand Rayleigh instability; otherwise, it would shatter into droplets [10]. Furthermore, temperature influences feed solution viscosity, with higher temperatures producing nanofibers with larger diameters [9]. In addition, an appropriate volatility of the solution feed is required, as highly volatile solutions might expedite jet drying, while lowly volatile solutions can result in insufficient drying and damage fiber formation [10]. Variable collector geometries can be used to change the fiber diameter, morphology, structure, and alignment [12,13]. The electric field can be manipulated by changing collector type, employing auxiliary electrodes, and regulating collector movement [16,17]. Table 1 summarizes the different parameters and factors that influence electrospun nanofiber production.

Because of their poor aqueous solubility, hydrophobic drugs can be more release-controlled than hydrophilic drugs, as hydrophilic drugs tend to diffuse into the medium rather than remain attached to the nanofibers, resulting in burst release [12]. Other factors that can cause an initial burst release include drug solubility in the polymer solution. A good interaction between the drug and the polymer results in a well-dispersed drug within the nanofibers, whereas a bad interaction causes the drug to accumulate on the nanofiber surface [10]. One of the main disadvantages of electrospinning methods is that they can result in an initial burst release due to the lack of a strong physical interaction between the drug and the polymer, resulting in the drug being dispersed at the nanofiber surface, as well as a high degradation rate. For example, nanofiber-based scaffolds require high drug concentrations to produce specific therapeutic responses, which can result in an initial burst release [5,12].

There are several approaches for reducing the occurrence of burst release, one of which is nanofiber crosslinking, which can be carried out using ultraviolet (UV) light, dehydrothermal (DHT) treatment, and chemical treatments such as glutaraldehyde [11]. Another approach is to incorporate inorganic nanocarriers into the nanofibers, such as silica-based nanoparticles, in which the drug is adsorbed on their surface before being dispersed in the polymer [9]. Initial burst release can also be controlled by adjusting the ratio of amorphous to crystalline composition, which will change the drug release kinetics and transport mechanism [10]. Coaxial electrospinning can also be used to produce core–shell structures, which prevent the initial burst release [9,10]. The composition and structure of nanofibers are crucial for achieving specific drug release profiles. Nanofibers for immediate release typically have a simple, homogenous structure that includes the drug and a polymer or polymer blend, whereas nanofibers for controlled release may have a similar structure but are significantly more complex. They are often composed of biodegradable or swellable polymers that degrade or swell in a controlled manner. Another approach for achieving controlled release is to use core–shell nanofibers [9,10], which can consist of multiple drug-loaded layers or an outer polymer layer that serves as a rate-controlling barrier for drug release. Controlled release can also be achieved by the sandwich approach (i.e., electrospinning different polymer solutions or melts consecutively to form sandwich-type nanofiber meshes) or by modifying the bead diameter in the nanofiber structure. The drug release kinetics can be tailored by modifying nanofiber material parameters such as polymer type and concentration, nanofiber diameter, structure, porosity, and surface functionalization [13,20]. Table 2 summarizes the different parameters and conditions that influence drug release from nanofibers.

### 2.3. Materials Used in Electrospun Nanofiber Fabrication

Polymers commonly used in electrospinning for cancer therapy include poly-ε-caprolactone (PCL), polylactic acid (PLA), polyethylene glycol (PEG), poly(L-lactide) (PLLA), polylactic-co-glycolic acid (PLGA), polyurethane (PU), polyvinyl alcohol (PVA), polyethylene oxide (PEO), polyvinylpyrrolidone (PVP), and natural polymers like cellulose, chitosan, hyaluronic acid, collagen, and peptide (Table 3). The type of polymer used is determined by the desired therapeutic effect and application. Formic acid, dichloromethane (DCM), hexafluoroisopropanol (HFIP), dimethylformamide (DMF), acetic acid, ethanol, trifluoroacetic acid (TFA), tetrahydrofuran (THF), and distilled water are all common solvents used in electrospinning [14]. The choice of polymer and solvent has significant effects on nanofibers. Higher polymer molecular weights produce a more homogeneous shape. The use of two different solvents resulted in a fibrous, highly porous structure [27]. In terms of polymer hydrophilicity, hydrophobic polymers are preferred over hydrophilic polymers that require rapid release kinetics. Increased polymer concentration results in a larger nanofiber diameter, whereas increased polymer conductivity results in a lower nanofiber diameter [9,10].

## 3. Forcespinning and Forcespun Nanofibers

### 3.1. The Concept of Forcespinning

Forcespinning is not a new industrial technology. For nearly 50 years, this technology has been extensively used to produce glass fibers (commonly known as fiberglass). However, using this technology to produce polymeric fibers, particularly polymer nanofibers, is relatively recent. The appeal of fiber-based composites in cancer therapy stems from their advantageous characteristics, such as high aspect ratios, large surface areas, and various morphologies [36]. Electrospinning, a popular technology for generating non-woven fibers, enables the production of fibers spanning in diameter from hundreds to thousands of nanometers. This technology is one of several approaches to achieving the desired characteristics for cancer applications [6,18]. On the other hand, the limitations of traditional electrospinning techniques, including material constraints, sluggish production rates, and high costs, have fueled advancements in nanofiber production. One promising advancement in this field is forcespinning, commonly known as centrifugal spinning, which eliminates the reliance on electric fields in favor of centrifugal forces [37]. By integrating centrifugal forces with various spinneret configurations, forcespinning offers a versatile approach that expands material options beyond the constraints of traditional methods, notably allowing for both conductive and non-conductive solutions [38]. Unlike electrospinning, where an electric field drives the process, forcespinning utilizes centrifugal force as its primary driving force. In this technique, a polymer solution or melt is contained within a rotating spinning head. As the rotational speed increases, the centrifugal force surpasses the surface tension, ejecting a liquid jet from the nozzle. This jet undergoes stretching and is deposited onto a collector, forming nanofibers as the solvent evaporates during the stretching phase [39]. Various factors, such as rotational speed, nozzle configuration, collection parameters, humidity, and temperature, influence the resulting nanofiber geometry and morphology [39]. Additionally, forcespinning offers flexibility by enabling the use of high-temperature solvents through spinneret heating. Notably, solid materials can be liquefied and spun without requiring preparatory chemical treatments, eliminating the need for solvent recovery [40].

**Figure 3:**
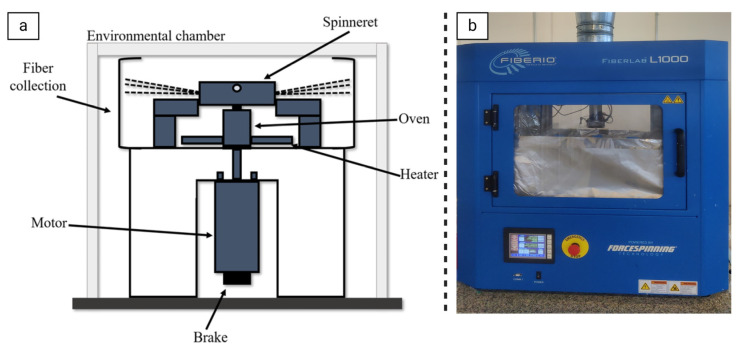
(**a**) A schematic illustration of the forcespinning system. Created with Microsoft PowerPoint; (**b**) a digital image, captured at Technologico de Monterrey in Monterrey, Mexico, of the forcespinning FiberLab™ L1000 (FibeRio^®^, Mcallen, TX, USA).

The forcespinning system, as depicted in Figure 3, comprises several integral components, including the spinneret, thermal system, collection devices, environmental chamber, control system, motor, and brake [40]. The control system operates the system according to user-specified speed and temperature set-points through a programmable logic controller, monitoring, and control. Additionally, the controller interfaces with a computer for remote process management. Within the chassis assembly, the motor and oven are housed, with the spinneret affixed to the shaft’s end via a threaded coupling, and the brake serves to halt motor operation during power failures and aids in spinneret installation. The thermal system incorporates both heating elements for material melting and thermo-electric coolers for low-temperature operations. The environmental chamber facilitates the maintenance of non-ambient conditions such as vacuum, inert gas, and aseptic environments, doubling as a safety enclosure for operators. In essence, the process involves depositing material, either as a solution or granular solids melted within the spinneret, which is then spun to produce nanofibers [41]. Key control parameters affecting fiber diameter include spinneret selection, nozzle configurations, material rheology, rotational speed, temperature, collection system design, and environmental conditions [39]. 

### 3.2. Factors Affecting Forcespun Nanofiber Fabrication

The forcespinning process commences by loading a polymer solution or a melt into a specially designed spinneret. Key polymer solutions and machine parameters are detailed in Table 4. During nanofiber production, rotational forces draw the polymer solution from the orifice. Key parameters include spinneret angular velocity, orifice radius, polymer viscoelasticity (e.g., viscosity and relaxation time), surface tension, evaporation rate, humidity, temperature, and distance of the spinneret orifice to the collector [37,39]. The rate of solvent evaporation affects polymer viscosity and elasticity. For polymer solutions, the compatibility of the polymer and solvent determines the ideal concentrations for producing nanofibers of the desired structure and characteristics [40]. Similarly, for melt forcespinning, selecting the optimal temperature is critical for making a viscosity suitable to nanofiber production. To form a polymer jet, rotational forces must exceed the solution’s or melt’s surface tension. Excessive forces can cause jet breakup and bead formation, particularly at low spinneret angular velocities or low viscosities [42]. Controlling viscosity is essential for successful fiber formation. High viscosity may hinder fiber drawing forces, while low viscosity can lead to bead formation. Additionally, low viscosity can result in beaded nanofibers due to reduced chain entanglement. Viscoelastic properties cause phenomena such as extrudate swelling, increasing jet diameter as it exits the orifice. These properties also lead to instabilities as the fiber shrinks and the solvent evaporates [40]. The evaporation rate of the polymer solution affects the fiber diameter. Rapid evaporation during jet acceleration reduces fiber diameter. Insufficient evaporation can lead to fiber merging into a thin film, while excessive evaporation disrupts jet elongation, producing large-diameter fibers [42]. Orifice diameter influences nanofiber production. Smaller diameters reduce clogging and bead formation while decreasing the overall fiber diameter. Orifice orientation and geometry also affect fiber formation [42]. Unlike electrospinning, collector distance in forcespinning primarily affects solvent evaporation time, fiber spiraling, and collection method. Short distances prevent stretching, yielding larger-diameter fibers. Once a critical distance is surpassed, further increases minimally affect fiber diameter reduction [40]. Modeling fiber formation considers spinneret angular velocity, polymer viscoelasticity, collector diameter, orifice radius, and solvent evaporation rate [43]. High-speed photography provides insights into initial fiber formation and trajectory towards the collector, aiding parameter optimization for material properties, operating parameters, and machine components [44]. Understanding nanofiber formation mechanisms contributes to better process design and product quality.

### 3.3. Materials Used in Forcespun Nanofiber Fabrication

Several polymers have been used in forcespinning, with each serving a distinct biological purpose (Table 5). Due to their lack of inherent bioactivity, synthetic polymers such as Nylon-6 [61], PLA [62], PU [50], PCL [60,63], and PEO [64] are widely used in biomedical applications. PVP stands out among synthetic polymers for its widespread use in forcespinning due to its safety, non-toxicity, water solubility, biocompatibility, biodegradability, and FDA recognition [65]. PVP fibers have demonstrated promising outcomes in oral drug delivery systems. These fibers exhibited excellent entrapment efficiency and drug loading capacity, particularly for antibiotics [66]. Furthermore, PVP fibers loaded with antioxidants derived from mangosteen and garlic hold promise for a variety of therapeutic applications [67,68]. Nonetheless, different synthetic polymers have shown promise for forcespinning applications. For example, Kodali et al. [69] discovered that increasing rotational speeds when spinning with PCL produced more uniform fibers with less bead formation. Furthermore, higher speeds were associated with lower crystallinity in the fibers. The Young’s modulus of the spun fibers ranged from 3.5 to 6 MPa, and with increasing fiber diameter, both storage and loss moduli decreased. Notably, fibers collected at greater distances from the spinneret demonstrated superior mechanical properties compared to those collected at shorter distances. On the other hand, natural polymers are preferred in several biomedical applications due to their renewable nature, improved biocompatibility, and controlled degradability, and outperform their synthetic counterparts [70]. Polysaccharides, originating from plants (e.g., cellulose, starch, and xylan) and animals (e.g., collagen, chitosan, and hyaluronan), offer great advantages for biomedical applications and drug delivery [71]. These include safety, non-toxicity, digestibility, biocompatibility, biodegradability, and renewability. Among them, chitosan, a prominent animal-derived polymer, stands out for its polycationic nature, biocompatibility, biodegradability, non-toxicity, mucoadhesiveness, and anticancer properties [72]. Researchers have delved into a range of polymer-based forcespinning, encompassing materials synthesized from starch, gelatin, and chitosan-pullulan blends. According to research, changing the amylopectin-to-amylose ratio in starch influences fiber properties such as surface morphology and topography [73]. Gelatin-based forcespinning exhibits higher thermal stability than that produced via electrospinning methods. Furthermore, combining polymers like chitosan and pullulan with natural extracts results in fibers with long, homogenous structures that have enormous promise in biological applications [74].

## 4. Methods of Drug Encapsulation and Release from Nanofibers

Electrospun nanofibers have been shown to be promising nanocarriers with high drug loading capacity and encapsulation efficiency, making them an excellent candidate for drug delivery. There are several methods used for the fabrication and drug encapsulation of electrospun nanofibers, including conventional electrospinning, melt electrospinning, coaxial electrospinning, emulsion electrospinning, blend electrospinning, and colloid electrospinning. These different methods provide versatility in fiber fabrication, enable the usage of a wide range of biocompatible and biofunctional materials, and diversify electrospun nanofiber applications in various biomedical fields, such as drug delivery and cancer therapy [24].

Drug encapsulation can occur both during and after electrospinning. There are various approaches for drug encapsulation. In the case of simple electrospinning, drug encapsulation entails solubilizing the drug in the polymer solution, which is governed by the solubility of the drug. In the case of coaxial electrospinning, core–shell structures are produced [84]. It uses a coaxial double-nozzle spinneret to blend two polymer solutions that would otherwise be incompatible by solubility [84]. In the case of emulsion electrospinning, which can combine both hydrophilic and hydrophobic drugs, the polymer is dissolved in an organic solvent while the drug is dissolved in another solvent. The two immiscible solutions are subsequently emulsified using a surfactant or a blend of surfactants [85].

Drug release from electrospun nanofibers is primarily caused by drug diffusion, drug dissolution, drug adsorption/desorption, and polymer erosion/degradation. In general, drug release is controlled by diffusion for non-degradable polymers [86], degradation for fast degradable polymers [87], and a combination of diffusion and degradation for slowly degradable polymers [88]. The polymer composition, polymer crystallinity, drug solubility in polymer, drug distribution in nanofiber, drug-polymer interaction, and fiber size and morphology all have significant effects on drug release kinetics from electrospun nanofibers [24]. The formulations of electrospun nanofibers generally exhibit typical biphasic drug release kinetics, starting with a burst release and progressing to a sustained release. Drug enrichment takes place at the fiber surface during the electrospinning of blended drugs and polymers, producing a high burst release. Core–sheath structures prepared by coaxial and triaxial electrospinning have been developed to better control drug release; the sheath barriers allowed for a controlled release [84]. The core–sheath structures exhibit zero-order kinetics with no burst release. This makes it an ideal release system for controlled drug release over an extended period of time [89]. Several mathematical models have been employed to predict the drug release mechanism, including zero-order kinetics, first-order kinetics, the Higuchi model, the Hixon–Crowell model, the Korsemeyer–Peppas model, the Peppas–Sahlin model, and the Weibull model [90,91]. The encapsulated drug in the polymeric nanofiber matrix is released through diffusion and matrix erosion. A diffusion-based mechanism transports drug molecules from higher-concentration to lower-concentration mediums, resulting in controlled drug release. Polymer erosion/degradation of the polymeric nanofiber matrix occurs in two ways: physically and chemically [24]. Bioactive polymers used in nanofibers are degraded via oxidation, hydrolytic cleavage, or enzymatic cleavage of bonds into small monomers or oligomers. Hydrophobic polymers erode by transforming into small water-soluble molecules as a result of backbone cleavages, whereas insoluble polymers erode via hydrolysis and ionization of the pendant group [24].

## 5. Methods of Functionalization and Surface Immobilization of Nanofibers

Nanofibers need to be functionalized in order to increase their characteristics and suitability for biomedical applications targeting cancer. In fact, functionalization can enhance the inherent characteristics of nanofibers or introduce new features. Functionalization of nanofibers is the process of modifying the nanofiber surface by introducing specific surface functional groups to fix and immobilize functional molecules, such as proteins, peptides, enzymes, and growth factors, on the nanofiber surface. This process helps improve their biocompatibility, increases cell adhesion, and enhances their active tumor-targeting capacities. Functionalization and surface immobilization can be employed when drug degradation is expected due to the use of strong solvents during drug–polymer mixing or when the drug has solubility issues. This can be performed by immobilizing the drug physically or chemically [92,93].

The physical surface functionalization involves plasma treatment methods, grafting-to and grafting-from methods, and layer-by-layer (LbL) assembly [94,95,96]. The plasma treatment method is used to modify the nanofiber surface without affecting its bulk properties by introducing functional groups such as –OH, –COOH, and –NH_2_ on their surface, resulting in enhancing their hydrophilicity and biocompatibility [95]. The grafting-to and grafting-from methods involve the covalent attachment of polymers or biomolecules to the nanofiber surface, leading to improved mechanical properties and bioactivity [97]. Layer-by-layer (LbL) assembly is performed by alternately depositing an oppositely charged polyelectrolyte on the nanofiber surface, which may allow for controlled drug loading and release and enhance cell adhesion and proliferation [94,96].

The chemical surface functionalization methods involve wet chemical treatment, silanization, and Steglich esterification [98,99]. In the wet chemical treatment method, chemicals such as strong acids, bases, or oxidizing agents are used to modify the nanofiber surface. This method can introduce functional groups to the treated nanofibers and improve their wettability, but it may accidentally cause structural damage [98]. The silanization method involves the reaction of silane coupling agents (silicon-based chemicals containing two sides of reactive functional groups, namely, inorganic groups, such as methoxy, ethoxy, and acetoxy, and organic groups, such as amino, methacryloxy, and epoxy), with the hydroxyl groups on the nanofiber surface, which allows for the covalent immobilization of polymers or biomolecules. It also enhances their biocompatibility and cell adhesion [99]. The Steglich esterification method uses N,N′-dicyclohexylcarbodiimide (DCC) and 4-dimethylaminopyridine (DMAP) to activate carboxyl groups. It enables the covalent attachment of polymers or biomolecules to the nanofiber surface and improves their mechanical properties and bioactivity [99].

Nanofibers with surface-immobilized functional groups can be developed by changing the nanofiber surface after production. Physical immobilization causes a burst release due to the electrostatic interactions, hydrogen bonding, hydrophobic contacts, and van der Waals interactions between the drug and nanofibers. These interactions are undesirable for cancer therapy, which requires controlled release profiles. While chemical immobilization can result in controlled release profiles, chemical immobilization can be achieved using a variety of methods, including polymerization, chemical conjugation, and mineralization [94,96].

## 6. Light-Responsive Smart Nanofibers as an Intelligent Stimuli-Responsive Nanocarrier

The ideal smart nanofiber formulations for drug delivery should be spatially and temporally controlled. Nanofibers are often delivered in dosage forms through a local delivery system. As a result, drugs are only released at the targeted site, reducing systemic exposure. Drug delivery can be spatially controlled by applying nanofibers at the targeted site using invasive or non-invasive methods. In earlier studies, the temporal control of drug release from nanofibers was primarily governed by drug diffusion rates, drug dissolution rates, drug physical desorption rates, and polymer degradation/erosion rates [92,93,94]. Recent studies have focused on developing activation and feedback properties for nanofibers that can initiate and regulate drug release over time. Such nanofibers are referred to as smart nanofibers because they typically contain a specific molecule experiencing physicochemical changes in this activation-modulated or feedback-regulated system. This system automatically responds to changes in environmental parameters such as light, which can modify the drug release rate based on prognostic markers [100].

### 6.1. Concepts and Mechanisms of Light-Responsive Smart Nanofibers

**Figure 4:**
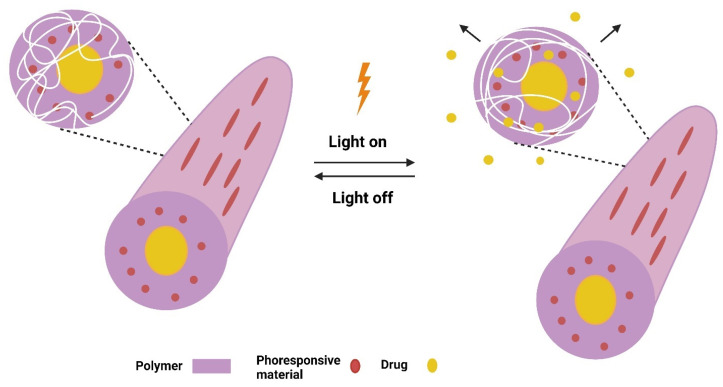
The functioning principle of light-responsive stimuli nanofibers. Created with BioRender.com.

Light is a tool for on-demand drug delivery owing to its controllability and customization. It has several advantages over other stimuli, including providing a wide range of effective and relatively safe stimulus levels; triggering various molecular processes orthogonally and sequentially; and being localized to specific sites within human tissues and organs to elicit a particular biological response, i.e., spatiotemporal control over the therapeutic window to minimize off-target activity and maximize therapeutic efficacy [100,101]. Photoresponsive materials are smart materials designed for precise tumor targeting since they can deliver light instantly and with high spatiotemporal accuracy via an on/off control mode. Light-responsive smart nanofibers have distinct functional properties that allow them to release their cargos when exposed to specific light wavelengths such as ultraviolet (UV, 200–400 nm), visible (400–700 nm), or NIR (700–1000 nm), resulting in high drug targeting efficiency and spatiotemporal control over drug release. They are primarily developed by incorporating photoresponsive materials into polymeric nanofiber matrices (Figure 4) [100,101]. In fact, UV light is the most widely used radiation because it delivers enough energy to start the vast majority of photochemical reactions. However, there are some disadvantages to employing UV light, including tissue penetration depth and phototoxicity. As a result, UV-responsive smart nanofibers have relatively few clinical applications. In turn, visible light can generate more energy; however, there are some limitations that limit its therapeutic applications, such as the limited tissue penetration depth of visible light and the ability of endogenous fluorophores, such as melanin and hemoglobin, to absorb visible light. In contrast, because of the limited attenuation and refraction caused by endogenous chromophores and proteins, NIR light penetrates deeper into tissues [100,101].

Light-responsive smart nanofibers can be developed in two distinct ways: (1) by covalently attaching groups of photoresponsive materials to the polymer, either as side chains or in the main chain, and (2) by non-covalently incorporating photoresponsive materials into the polymer matrix via blending. These smart nanofibers may respond to light exposure in a variety of ways, including photoisomerization, photochromism, photocatalysis, and wettability changes [102,103,104].

**Figure 5:**
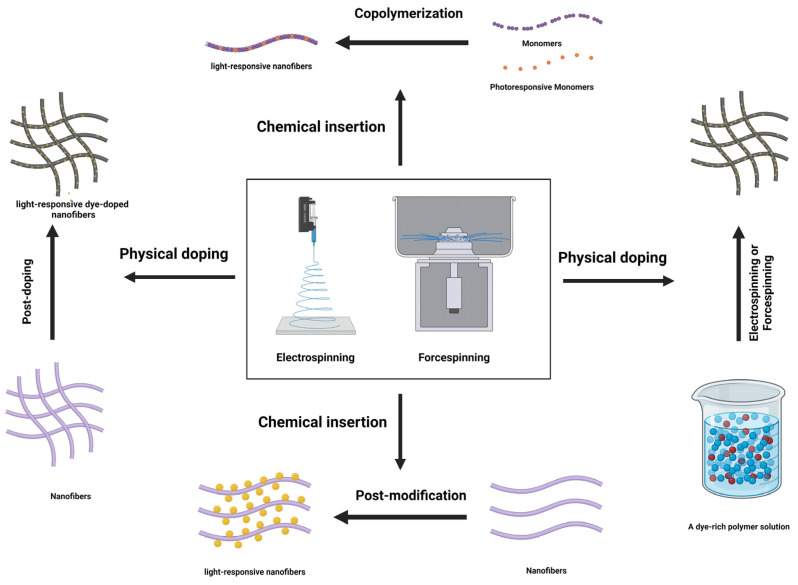
A schematic illustration of photo-responsive electrospun nanofiber preparation methods. Created with BioRender.com.

Chemical insertion and physical doping are the two most common methods for developing light-responsive nanofibers (Figure 5). In the first method, the groups of photoresponsive materials are introduced into polymer chains by several chemical methods, resulting in high stability for smart moieties [105]. However, the second method provides a simple and straightforward preparation method in which photoresponsive dyes are physically deposited inside or on the surfaces of nanofibers [106]. The chemical insertion method can be classified into two categories: copolymerization of photoresponsive monomers with other monomers [105] and post-modification of nanofibers [107]. Photoresponsive moieties can be inserted into the polymer backbone as the main chain or pendant groups using various polymerization methods, such as free radical, atom transfer radical polymerization, reversible addition-fragmentation chain transfer, and so on. In order to do this, acrylic photoresponsive monomers must be synthesized. In the second method, smart dyes are introduced into polymer chains after nanofiber preparation by chemical post-modification methods such as hydrogen abstraction reactions. This method also necessitates functionalized photoresponsive dyes. Two approaches are used to develop photoresponsive nanofibers via the physical doping method. The most common approach is to combine dyes with a spinnable polymer solution [108]. However, several studies used the post-doping method, in which photoresponsive dyes are applied to the surface of nanofibers by immersing them in a dye-rich solution [109] or brushing such a solution onto the nanofibers [110].

**Figure 6:**
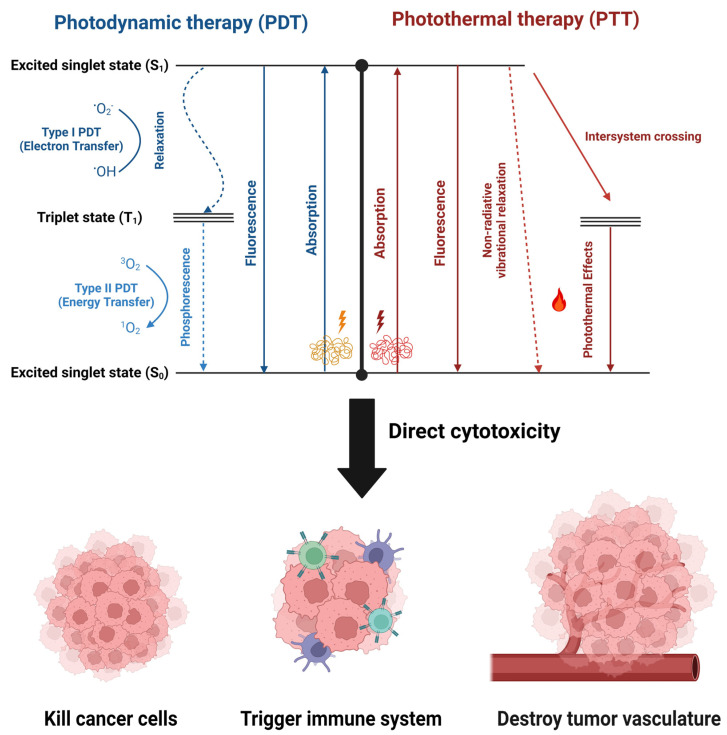
The mechanisms of photodynamic and photothermal therapies. Created with BioRender.com.

Photoresponsive materials have unique optical properties that enable them to convert light energy into heat, electrical stimuli, or even chemical reactions (e.g., *cis–trans* isomerization and open-closed ring transformation), which are utilized in the treatment of cancer. They usually consist of either organic molecules like chromophores or fluorophores or inorganic nanomaterials like carbon-based nanocarriers (e.g., carbon nanotubes) and metal-based nanocarriers (e.g., gold nanocages and gold nanorods), which act as agents for photothermal and photodynamic therapies [111,112,113]. Photodynamic therapy (PDT) uses light to activate photoresponsive materials, resulting in the generation of reactive oxygen species (ROS) in the form of free radicals (hydroxyl (HO•) and superoxide (•O_2_) or non-radicals (^1^O_2_), which are highly reactive oxidants that can damage tumor cells, whereas photothermal therapy (PTT) uses light to generate heat from plasmonic nanoparticles to kill tumor cells (Figure 6) [114]. 

As an example of light-responsive smart nanofibers encapsulating photochromic chromophores, Li et al. [115] developed NIR light-responsive electrospun nanofibers consisting of zwitterionic poly(2-methacryloyloxyethyl phosphorylcholine)-*b*-poly(ε-caprolactone) (PMPC-*b*-PCL), encapsulated with chromophore indocyanine green (IG), a photothermal agent, and doxorubicin (DOX), an anticancer drug for dual photothermal therapy and chemotherapy. Under mild NIR irradiation, IG converted NIR light into thermal energy, accelerating DOX release from the nanofibrous composite due to nanofiber softening, implying that drug release could be controlled and switched on/off via light-triggering. Furthermore, the light-induced thermal energy and release behavior contributed to increased cell lethality. The developed electrospun nanofibrous composite exhibited excellent antifouling properties and a high potential for combined chemotherapy and PTT in inhibiting cell growth, and served as an effective cancer treatment with low systemic toxicity. 

As an example of light-responsive smart nanofibers encapsulating plasmonic nanocarriers, Li et al. [116] developed NIR light-responsive electrospun nanofibers consisting of PNIPAM and mesoporous silica-coated gold nanorods (Au@SiO_2_) loaded with DOX, a photothermal drug carrier, and crosslinked by polyhedral oligomeric silsesquinoxanes (POSS) (termed PNIPAM-POSS-Au@SiO_2_). The true molecular dispersion of POSS with organic polymers, the photothermal effect of Au@SiO_2_, and the thermos-response of PINPAM were combined to endow the nanofibers with remarkably reversible volume phase transitions, which were demonstrated for the first time at the polymer molecular level. The drug release was found to be abundant and repeatedly accelerated by external NIR light, and the released DOX reduced the viability of human cervical cancer HeLa cells by 97%. Simultaneously, the nanofibers enabled model cell NIH3T3 fibroblast entrapment, adhesion, proliferation, differentiation, and cell release after NIR irradiation with no effect on cellular function. Another promising example is Haghighat Bayan et al. [117], who developed a light-responsive electrospun nanofibrous core–shell platform for on-demand drug release. Core–shell PVA-PLGA nanofibers with plasmonic nanoparticles were prepared by using coaxial electrospinning and electrospraying techniques. The prepared nanofibers had a hydrophilic PVA and Rhodamine-B (RhB) core with a hydrophobic PLGA shell decorated with gold nanorods as a plasmonic agent to induce on-demand drug release upon NIR irradiation. Because the PVA core was water-soluble, the PLGA shell played a key role in protecting it from structural damage. The drug was released from core–shell PVA-PLGA nanofibers using a NIR light. In comparison to the non-irradiated system, the RhB release from the activated system was successfully elevated in response to light stimuli. Furthermore, the nanofibrous core–shell platform demonstrated structural stability both before and after NIR light exposure, and plasmonic nanoparticles enhanced drug release and provided long-term stability.

Smart nanofibers were also exploited in the PDT field by introducing photosensitizers (PhS), which produce ROS when exposed to light and oxygen, leading target cells to die due to oxidative damage [118]. They have presented a viable option against various types of cancer due to their localized nature, which, when implanted in the tumor site, can target and augment the therapeutic efficacy of loaded PhS while reducing undesirable side effects, resulting in a lower PDT dose, increased bioavailability, and improved in vivo stability [119,120]. For example, Wu et al. [121] developed PLLA electrospun nanofibers containing purpurin-18 as a PhS that was cytotoxic to both the human hepatocellular carcinoma cell line (SMMC-7721) and the human oesophagal cancer cell line (ECA-109), with a reduction in cell viability of both cell lines after exposure to PDT irradiation. Another interesting example is Ma et al. [122], who developed electrospun nanofibers of PLLA/PEO polymers with 5,10,15,20-tetrakis (4-carboxyphenyl) porphyrin (TCPP) as a PhS. It demonstrated a burst release of TCPP within the first 7 h, followed by a sustained release over 72 h. TCPP-loaded PLLA/PEO nanofibers showed modest dark toxicity on human cancer cells (HeLa cells) but increased their death after radiation exposure, demonstrating that PDT kills cancer cells.

### 6.2. Development of Light-Responsive Smart Nanofibers

Human bodies are frequently exposed to light (natural and artificial light). The wavelength of light encountered in daily life ranges from 3000 nm for an infrared heater to 315 nm for ultraviolet light A (UVA) in sunlight. Ultraviolet (UV) light is ineffective for use in therapy below 315 nm because high-energy photons directly damage DNA [123]. Light-responsive smart nanofibers have shown substantial potential for tunable drug delivery and release to specified targets, as well as the capacity to turn on and off in response to light stimuli. For safety reasons, light-responsive smart nanofibers should be able to respond to light having a wavelength greater than 315 nm [123,124]. 

**Figure 7:**
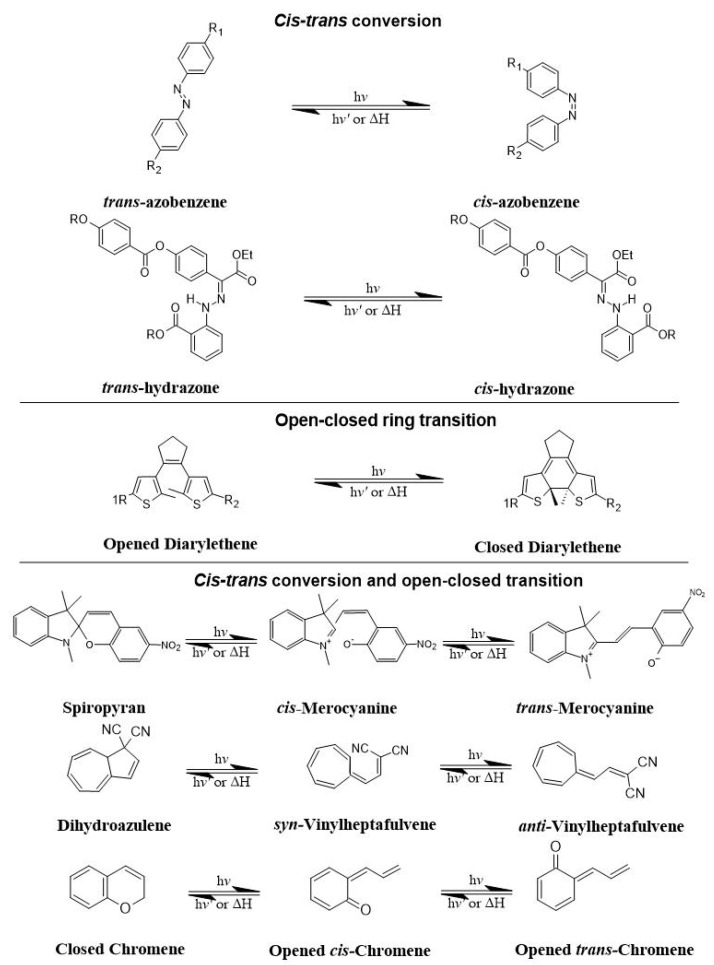
The most common molecular photoswitches and their mechanisms. Created with ACD/ChemSketch Software 2021.2.1 (Advanced Chemical Development, Inc., Toronto, ON, Canada).

Photoisomerization paves the way for the development of light-responsive smart nanofibers. There are two major classes of photoisomerization behaviors, including *cis–trans* conversion (as in the case of azobenzenes and their heteroaromatic analogues) and open–closed ring transition (as in the case of diarylethenes), which have been used in light-responsive smart nanofibers (Figure 7). Photoisomerization refers to molecular changes caused by light irradiation that changes their configuration, rearrangement of atoms or groups within the molecule, and chemical structure and properties. This reaction can be reversible or irreversible depending on the condition and structure of the compound, as observed in photochromic materials such as azobenzenes, diarylethenes, and spiropyrans, where light absorption induces reversible interconversion between the two isomeric forms of a molecule. These isomeric forms may differ in color and other characteristics, such as polarity, due to differences in absorption spectra, conformation, and charge presence. The isomerization process and properties of these materials can be tailored by adjusting light exposure parameters such as wavelength and intensity. Photochromic materials have recently received increased interest due to their use in light-responsive smart nanofibers. The photoresponsivity of these materials is based on photoisomerization of the constituent molecules, which commonly includes *cis–trans* conversion, open–closed ring transition, intramolecular proton transfer, and redox reactions [105]. For example, azobenzene, a well-known photoresponsive chromophore, experiences a photo-reversible *cis–trans* conversion. As one of the key photochromic compounds, the ring-closed, non-colored, and non-polar spiro (SP) form of spiropyran can be converted to the ring-opened, colored, and polar merocyanine (MC) isomer after light exposure [105].

**Figure 8:**
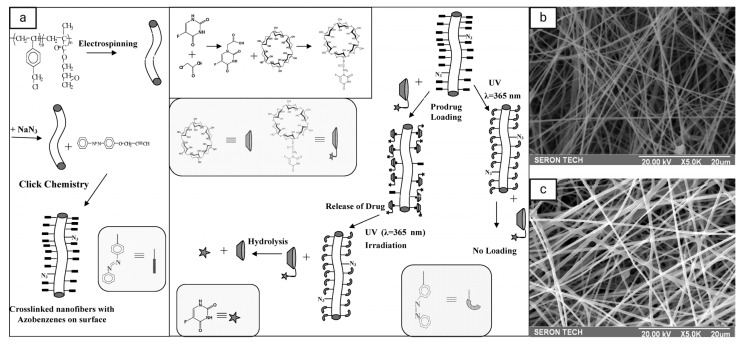
(**a**) A schematic illustration of the preparation of the cross-linked nanofibers of PVBC-*b*-PGMA with azobenzene groups on the surface (CNF_PVBC-b-PGMA_-Azobenzene), the synthesis of the CD and 5FU prodrug (α-CD-5FU), and the photoresponsive loading and release of the α-CD-5FU prodrug on the CNF_PVBC-*b*-PGMA_-Azobenzene surface by host-guest interaction; (**b**) SEM image of the as-synthesized CNF_PVBC-*b*-PGMA_-Azobenzene (with 74 VBC repeat units and 46 GMA repeat units in the copolymer molecule); (**c**) SEM image of the CNF_PVBC-*b*-PGMA_-Azobenzene after loading and photocontrolled release of the α-CD-5FU prodrug. Reprinted with permission from reference [125]. Copyright 2024 American Chemical Society.

One of the first attempts to use UV light-responsive smart nanofibers in drug delivery was by Fu et al. [125], who synthesized UV-triggered drug-release electrospun nanofibers using cyclodextrins (CDs) loaded with azobenzene, which switched from *trans*- to *cis*-form under UV light irradiation at a wavelength of 365 nm and then switched back under visible light (Figure 8). First, they prepared cross-linked nanofibers with azido groups on the surface using electrospinning of the block copolymer of vinyl benzyl chloride (VBC) and glycidyl methacrylate (GMA) (termed PVBC-*b*-PGMA), prepared by consecutive reversible addition fragmentation chain-transfer (RAFT) polymerization and subsequent reaction with sodium azide, then introducing of UV-sensitive groups on the nanofiber surface in between azide groups and 4-propargyloxyazobenzene (PAB), and finally loading of the anticancer prodrug 5-fluorouracil (5FU) in the form of α-CD-5FU to the nanofiber surface. The resultant nanofibers showed efficient and on-demand controlled drug release due to their high environmental stability and large effective surface area for drug loading.

**Figure 9:**
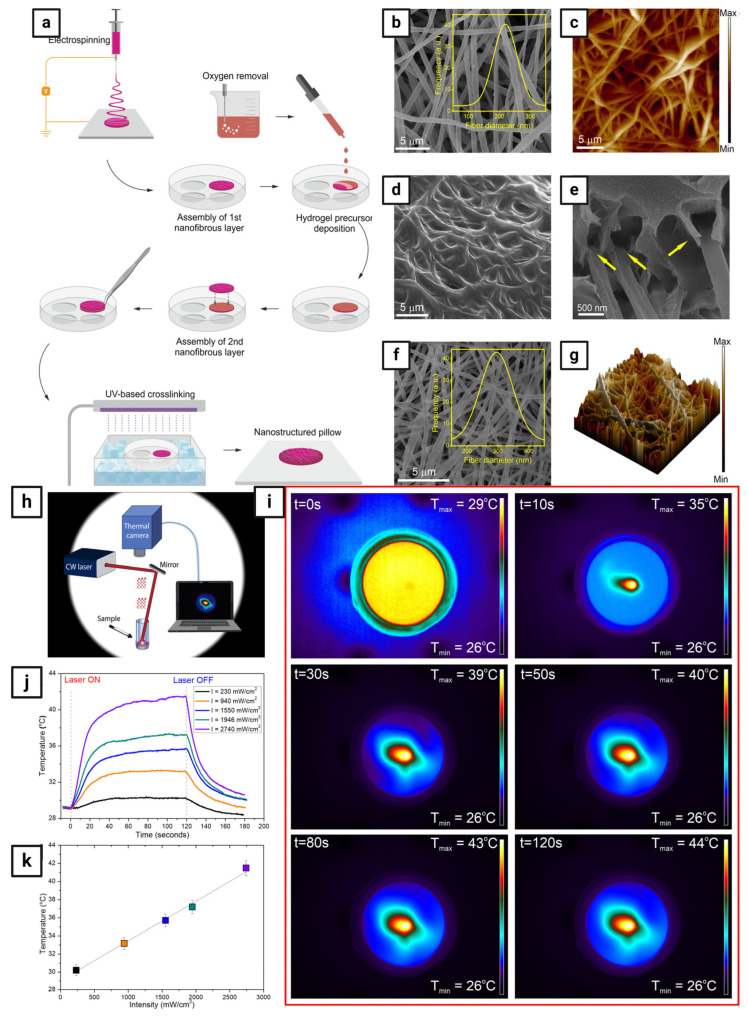
(**a**) A sheme showing the pillow preparation process, starting from the electrospinning of PLLA/RhB nanofibers to the free-radical polymerization of the plasmonic hydrogel; (**b**) SEM morphology of spun PLLA/RhB nanofibers with a fiber diameter of 210 ± 53 nm; (**c**) the surface morphology of PLLA/RhB nanofibers observed with AFM shows an extremely rough nanofibrous material (the z-scale is equal to 5 μm); (**d**) top view of the PLLA/RhB nanofibers disassembled from the pillow showing the interpenetration of nanofiber pores with the hydrogel at the hydrogel-fiber interface; (**e**) cross-section of the pillow showing the nanofiber anchorage in the hydrogel mass (the yellow arrows indicate the anchoring points); (**f**) SEM morphology of PLLA/RhB nanofibers after pillow preparation and UV irradiation (the external side unconnected with the hydrogel) with the fiber diameter distribution showing an average fiber diameter of approximately 300 ± 67 nm; (**g**) three-dimensional AFM image of the final pillow surface showing a typical porous nanostructure (25 μm × 25 μm; z-scale: 5 μm); (**h**) scheme presenting an experimental setup where the laser beam runs from top to bottom, irradiating the pillow in a vial filled with water, which was observed with a thermal camera while recording images directly on a computer; (**i**) thermographic images captured at different times during the laser irradiation (intensity: 2740 mW/cm^2^) of a floating single pillow; (**j**) temperature-time plots for various intensity values of the laser beam; (**k**) linear correlation between the maximum temperature detected and the intensity of the laser beam. Reprinted with permission from reference [128]. Copyright 2024 American Chemical Society.

On the other hand, NIR light can be used to generate heat for hyperthermia when combined with a photothermal agent. Carbon, gold, metal oxides, and sulfides are examples of photothermal agents that absorb and transform NIR light into heat [126]. Because of the low attenuation and refraction caused by endogenous chromophores, NIR light penetrates deeper into tissues. Although NIR light has the advantage of deeper tissue penetration and less damage to normal cells, only a few compounds can directly respond to it due to its low energy, which is insufficient to induce photochemical reactions. To address this issue, smart nanofibers capable of converting incident NIR light into UV light have been developed [114]. One of the first attempts to use NIR light-responsive smart nanofibers in drug delivery was by Nakielski et al. [127], who developed a nanofibrous system that can respond to NIR light and is made of a poly(L-lactide)-Rhodamine B-loaded nanofibrous material (PLLA/RhB), which encapsulated P(NIPAAm-co-NIPMAAm) hydrogel containing gold nanorods (AuNRs) (Figure 9). The mechanical contraction of the nanoplatform caused by the increase in temperature to 42 °C due to plasmonic hydrogel-light interaction resulted in the rapid repulsion of water from the inner structure, which passed through an electrospun membrane tethered to the hydrogel core. The combined effects of the rising temperature and water flow triggered the release of molecules from the nanofibers. To broaden the potential applications of the biomimetic platform, the photothermal responsiveness to attain the typical temperature level for performing photothermal therapy was developed. The on-demand drug model penetration into pig tissue demonstrated the efficiency of the nanoplatform in the on-demand controlled release of molecules, while the high biocompatibility confirms its potential for biomedical applications based on an NIR light-driven multitherapy strategy. Another leading example is Cheng et al. [128], who developed PEG-modified gold nanorod (PEG-GNR)-loaded nanofibers, which served as both a platform for PTT and a post-surgical physical barrier. Once released from the nanofibers, the GNRs were internalized by cancer cells and produced heat when exposed to NIR radiation, inhibiting cancer cell growth and death. 

### 6.3. Design of Smart Nanofibers with Photoresponsive Properties

Several designs have been reported for the preparation of nanofibers in general and light-responsive smart nanofibers in particular, such as monolithic design, core–shell design, and layer-by-layer assembly.

#### 6.3.1. Monolithic Design

Monolithic fibers have a single structure composed of polymers and drugs dispersed in a polymer solution, allowing drugs to be encapsulated before electrospinning in a single process, resulting in controlled release profiles [129]. Importantly, the physicochemical properties of polymers have a significant effect on the functioning and release rate of the encapsulated drugs due to direct interactions between polymers and drugs [130]. One important issue to consider while employing this design is the solubility of the drug. Drug molecules may migrate to the nanofiber surface due to insufficient solubility, resulting in burst drug release; however, maintaining an equilibrium between hydrophilic and hydrophobic molecules and polymers can help overcome this challenge [131]. Examples of PTT-loaded monolithic nanofibers for cancer therapy: Obiweluozor et al. [132] developed a single fibrous nanoplatform that combined PTT and chemotherapy and investigated its efficacy on CT-26 carcinoma cells. This fibrous nanoplatform contained lethal polydopamine nanospheres (PDA-NPs). NIR absorption bands have been observed in PDA-NPs, and the subsequent rapid heating has the ability to kill cancer cells. Another interesting example is Zhang et al. [133], who developed electrospun DOX-loaded PLLA nanofibers and multiwalled carbon nanotubes (MWCNTs) to integrate PTT and chemotherapy into a single compartment. Effective control of the NIR light irradiation maintained the MWCNTs in the vicinity of the tumor, allowing for repeated localized heating. Furthermore, it displayed excellent light-switch on-and-off control over the drug release at the tumor site. Zhao et al. [126] employed blend electrospinning to produce light-responsive nanofibers containing DOX and a photothermal transforming agent (MoS2) to treat postoperative tumor recurrence. When subjected to an 808 nm laser in vivo, the synthesized chitosan/PVA/MoS2/DOX nanofibers achieved a photothermal conversion efficiency of 23.2%.

As an example of PDT-loaded monolithic nanofibers for cancer therapy, Severyukhina et al. [134] employed chitosan and PEO biodegradable polymers to develop nanofibers loaded with PhS that had a high absorbance in the NIR region. The release showed a burst pattern during the first 24 h, which was related to the release of physically adsorbed PhS during the swelling of the nanofibers, followed by sustained release caused by the slow dissociation of the chitosan-PhS complexes, with a higher overall release at pH 7.4 compared to pH 5.5. The authors found that the toxicity of the nanofibers increased with increasing PhS content. However, the radiation exposure led to a substantial reduction in the metabolic activity (>90%) of carcinoma cells, whereas osteoblasts were resistant to the photodynamic effect, highlighting the phototoxic effect on malignant tissues. Another interesting example is van Hest et al. [135], who developed PEGylated PhS-peptide nanofibers that selectively target the tumor and exhibit acid-induced enhanced _1_O^2^ generation, cellular uptake, and PDT efficacy in vitro, as well as rapid accumulation inside the tumor, allowing long-term tumor imaging capacity and effective PDT in vivo, as well as prognostic monitoring of the efficacy of PDT in vivo, which could potentially guide cancer treatment. Liu et al. [136] studied enhancing the water solubility of PhS by developing a water-soluble porphyrin derivative, Ac-Asp-Glu-Val-Asp-Asp-TPP (Ac-DEVDD-TPP), to enhance the apoptosis of oral squamous cell carcinoma (OSCC). Upon caspase-3 (Casp3, an activated enzyme during apoptosis) cleavage and laser irradiation, Ac-DEVDD-TPP was converted to D-TPP, which spontaneously self-assembled into porphyrin nanofibers, accompanied by 1.4-fold and 2.1-fold _1_O^2^ generations in vitro and in cells, respectively.

#### 6.3.2. Core–Shell Design

Core–shell nanofibers consist of an inner core surrounded by an outer shell that serves as a protective covering, protecting drugs in vivo and allowing for more sustained and controllable drug release profiles [137]. They are typically produced by coaxial electrospinning, which involves drawing liquid into the nanofiber with an electrical charge, resulting in a core–shell structure [138]. Coaxial electrospinning enables fine control over core and shell structures, resulting in tailored characteristics and functions as well as increased encapsulation efficiency and drug loading capacity. Core–shell nanofibers have a lesser chance of initial burst release when compared to monolithic nanofibers [124,131]. As an example of PTT-loaded core–shell nanofibers for cancer therapy, Park et al. [139] produced core–shell electrospun nanofibers from PCL nanofibers containing DOX in the shell and a phase-changeable fatty acid in the core, as well as gold nanocages (AuNCs). The exceptional photothermal capacity and photostability of AuNCs allowed for recurring, substantial heating of the nanofibers to temperatures capable of inducing hyperthermia when NIR light was turned on. Furthermore, the heat generated by the NIR light melted the fatty acid, allowing the drug molecules to be released instantly from the nanofibers. Another interesting example is Azerbaijan et al. [140], who incorporated graphene oxide/gold nanorods (GO/AuNRs) and paclitaxel (PTX) into poly(tetramethylene ether)glycol-based polyurethane (PTMG-PU) (core) and chitosan (shell) nanofibers. The PTT/chemotherapy strategy explored the viability of using the prepared nanofibers as a pH/temperature dual-responsive nanocarrier for controlled PTX release against A549 lung cancer. According to the in vivo studies, the PTT/chemotherapy strategy offered an optimal therapeutic effect on tumor inhibition while having no effect on normal tissues. Li et al. [141] incorporated Rose Bengal (RB) and carmofur (CAR) into nanofibers composed of Eudragit L100-55 polymer (shell) and RB-loaded hydroxypropyl methylcellulose (HPMC) (core). The cytotoxicity of human dermal fibroblast (HDF) and colon cancer (Caco-2) cells was investigated to determine the effect of light on cell death. The RB-loaded nanofibers resulted in good vitality (about 80% for both cell types) in the absence of light but considerably increased toxicity (30–50%) with light. 

The use of core–shell nanofibers in PDT cancer applications has not been thoroughly studied. However, due to their distinct architectures, core–shell nanofibers are projected to exhibit less burst release in the initial stages while promoting controlled release of encapsulated PhS than monolithic nanofibers, making them a feasible choice for drug delivery in PDT cancer therapy [118]. A promising example is Kabay et al. [130], who produced stimuli-responsive nanoparticle-nanofiber hybrids (NNHs) by electrospinning photoresponsive natural melanin nanoparticles (MNPs) into a biocompatible PCL nanofiber matrix (MNPs-PCL), resulting in monolithic and core–shell structures. PDT with UV-A irradiation on monolithic and core–shell NNHs resulted in up to 34% and 37% malignant melanoma cell killing, respectively.

#### 6.3.3. Layer-by-Layer Assembly

Another promising method for developing nanofibers is layer-by-layer (LbL) assembly, which involves depositing alternating layers of oppositely charged materials on the surface of nanofibers after electrospinning using a variety of processes, including immersion, spin, spray, electromagnetic, and fluidics methods [124]. This method increases formulation flexibility, allowing for the use of different drugs, polymers, and solvents. For example, Singh et al. [142] designed a nanofiber system containing positively charged DOX and camptothecin (CPT) to release drugs sequentially in response to light. After being inserted into the nanofibers, gold nanorods with NIR absorbance were subjected to NIR light, causing the nanofibers to heat locally via plasmon resonance and cause controlled release through swelling and shrinking. The first layer was composed of linear polyethyleneimine (a polycation) and poly(sodium 4-styrene sulfonate) (a polyanion), acting as a simple compensating layer for the negatively charged layer above it. After being exposed to NIR radiation, CPT was released in a controlled pattern, followed by DOX. Another interesting example is Tiwari et al. [143], who developed implanted, targeted multifunctional nanofibers based on PDA for highly effective photothermal chemotherapy. PCL-DOX nanofibers were produced by simple electrospinning, and the surface was modified by chemically polymerizing PDA at various concentrations. This demonstrated dual responsiveness to pH and NIR, resulting in improved drug release in an acidic medium relative to physiological pH settings (pH 7.4), which was further augmented by NIR exposure. When subjected to an 808 nm NIR laser, the resulting membranes demonstrated remarkable stability and photothermal behavior. Table 6 summarizes the different designs of smart nanofibers with photoresponsive properties.

### 6.4. Characterization of Light-Responsive Smart Nanofibers

Several characterization techniques have been used to study the morphological, physicochemical, and photophysical properties of light-responsive smart nanofibers. Among these characterization techniques are the following:

#### 6.4.1. Structure, Morphology, and Porosity

Scanning electron microscopy (SEM) is commonly used to evaluate the structure, morphology, and porosity of nanofibers; however, multi-layer and core–shell nanofibers are among the most complex nanostructures evaluated using transmission electron microscopy (TEM) [145]. Confocal laser scanning microscopy (CLSM) and fluorescence microscopy are effective methods for studying nanofibers containing luminous polymers or drugs [146]. Porosity, defined as the empty spaces between nanofiber components, can be measured by SEM, nuclear magnetic resonance (NMR), liquid extrusion porosimetry, mercury intrusion porosimetry, and capillary flow porosimetry [147].

#### 6.4.2. Crystal Structure and Chemical Composition

The crystal structure and chemical composition of nanofibers are evaluated using X-ray diffraction (XRD) and Fourier-transform infrared spectrometry (FT-IR) to examine changes in the crystal structure of drugs and polymers [148]. In addition, FTIR can be used to characterize the interactions between nanofibers and their components. Furthermore, differential scanning calorimetry (DSC) can be used to evaluate the solid-state characteristics of active materials as well as the efficacy of encapsulating crystalline molecules [131].

#### 6.4.3. Mechanical Properties

Mechanical characterization involves a variety of techniques, including atomic force microscopy (AFM), microscale tension testing, resonance frequency analysis, bending tests, and nano-indentation [145].

#### 6.4.4. In Vitro Release and Ex Vivo Cell Line Studies

It is critical to understand the release mechanism and kinetics of light-responsive smart nanofibers. The simplest method to determine the release mechanism and kinetics of these systems is to change the light or wavelength settings and study the drug release pattern in response to light stimuli. Furthermore, understanding drug release mechanisms and kinetics helps predict the in vivo performance of the system and simulate in vivo release patterns [148]. Ex vivo cell line models are used to evaluate the efficacy and targeting functions of the produced nanofibers, as well as cell internalization for successful cancer therapy through cell viability and cytotoxicity testing [149].

### 6.5. Applications and Clinical Status of Light-Responsive Smart Nanofibers in Cancer Therapy

Cancer is a group of diseases that can occur in almost any tissue or organ of the human body when abnormal cells multiply uncontrollably, invade surrounding healthy tissues, and spread to other organs [150]. When the body requires new cells, healthy cells grow and spread in accordance with normal human physiology, while aging or damaged cells undergo apoptosis to be replaced by younger cells. However, this well-organized system is periodically disrupted, allowing abnormal or damaged cells (cancer cells) to reproduce and spread uncontrollably. This makes it challenging for the body to operate normally, impacting the area where cancer cells multiply and causing tumors to develop [150]. Cancer cells can spread throughout the body via the circulatory and lymphatic systems, resulting in metastases, which are the major cause of cancer-related fatalities [150]. Cancer can occur for a variety of reasons, including different mutations caused by genes, lifestyle choices, or exposure to cancer-causing substances [150]. Currently, the most common cancer treatment methods include surgery, chemotherapy, radiotherapy, and gene therapy, with chemotherapy remaining the primary strategy for the clinical treatment of diverse cancer types [151]. Because of the lack of targeting, chemotherapy can harm normal tissue cells while killing cancer cells, resulting in severe toxic side effects [151]. As a result, some researchers have developed stimuli-responsive smart nanofibers for cancer treatment that can respond within the tumor microenvironment to a stimulus (such as ROS) or an external stimulus (such as light) at the tumor site to release drugs, improve drug efficacy in killing tumor cells, and reduce harm to normal tissues [118,119]. In general, nanofibers have been used for cancer diagnosis and treatment [9,12]. Nanofibers can play a part in cancer diagnosis through cancer cell capture and detection, which is an active area of research [9,10,11,12]. Moreover, nanofiber-based biosensors have also been introduced to measure cancer biomarkers [12]. There are many advantages that electrospun nanofibers can introduce in nanofiber-based anticancer systems; these include the feasibility of drug loading during spinning methods, the large surface-to-volume ratio, the unique architecture providing porosity and interconnectivity, and the ability to manipulate drug release through adjustments in material and polymer composition [9,10,11,12].

Following are selected examples of light-responsive smart nanofibers in cancer therapy: Choi et al. [152] prepared a nanofibrous system capable of synergizing cancer therapy in addition to controlling and targeting drug release using electrospinning. It was composed of a PLGA polymer. The core contained the anti-cancer drug DOX, while the shell contained gold nanorods acting as a photothermal agent. The photothermal agent generated heat upon exposure to NIR light, elevating the temperature of the nanofibers. Upon exceeding the polymer glass transition temperature (Tg), mobility of the polymer chains occurred, widening the free volume inside the shell and accelerating the drug release. During the NIR light-off period, drug release terminated due to the immobility of the polymer chains, allowing targeted and enhanced anti-cancer drug effectiveness. The effectiveness and potency of the prepared nanofibers were tested on SKBR3 human breast cancer cells. Chen et al. [153] developed polyaniline-loaded PCL-gelatin nanofibers by electrospinning for the treatment of H22 hepatocellular carcinoma. Upon exposure to laser irradiation, polyaniline was capable of transforming the optical energy into heat energy. This led to the accumulation of NIR optical energy in the tumor cells for thermal excision of tumor cells. Li et al. [35] employed black phosphorus (BP) nanosheets as a light-responsive material embedded in electrospun nanofibers. The developed BP nanofibers prepared by electrospinning possessed a large surface area and high biocompatibility. The nanofiber photothermal properties led to the killing of the HepG2 cancer cells by increased temperature upon exposure to NIR radiation (808 nm, 2.5 W/cm^2^). This study demonstrated that electrospun nanofibers were capable of local treatment of cancer. Wang et al. [154] used electrospinning to create biopolymer nanofibers with Cu2S nanoflowers in their structure, which were then tested against skin cancer cells and demonstrated the ability to kill over 90% of tumor cells when subjected to NIR irradiation, reducing tumor growth in mice.

Despite the fact that light-responsive smart nanofibers have been proven to be an excellent platform for delivering and targeting anticancer drugs with minimal side effects, clinical trials in this field have yet to be done. On the one hand, the high external light stimulation required by this system could harm healthy tissues. Furthermore, it can be difficult to regulate the response parameters, resulting in drug leakage, serious side effects, and toxicities, as well as numerous drug leaks that endanger patient lives. Since the safety and toxicity of light-responsive smart nanofibers have not been extensively demonstrated, securing regulatory approval for clinical trials using these materials has been particularly problematic.

## 7. Light-Responsive Smart Multifunctional Nanofibers

Smart multifunctional nanocarriers enable the precise delivery of multiple drugs at the same time, improving therapy efficacy or overcoming tumor drug resistance. The distribution of multiple drugs also allows for the use of a variety of therapeutic approaches, such as chemotherapy and PTT or chemotherapy and PDT. The development of smart multifunctional nanocarriers with favorable characteristics has the potential to dramatically improve the efficacy of various therapeutic and diagnostic protocols. These smart systems function at the nanoscale and give new and powerful cutting-edge capabilities for cancer imaging, diagnostics, and therapy. Among these smart systems are light-responsive smart nanofibers with embedded nanoparticles, liposomes, or hydrogels. 

Nanofibers and nanoparticles are excellent candidates for targeted drug delivery in cancer therapy. However, nanofibers outperformed conventional nanoparticles because of their high reproducibility, biocompatibility, ease of manufacturing, high mechanical strength, and adjustability of the structural, morphological, and surface chemistry properties with simple tuning of the operating parameters, compared to conventional nanoparticles, which are susceptible to human error during the manufacturing process. Furthermore, conventional nanoparticles have a low-to-moderate drug loading capacity and exhibit an initial burst drug release, which impedes nanoparticle-based therapeutic commercialization [120]. Shan et al. reported this [155], using bovine serum album-loaded nanoparticles and nanofibers for comparison reasons. Nanofibers demonstrated more sustained drug release than nanoparticles, since an initial burst release of the drug adsorbed on the nanoparticle surface occurred, followed by a diffusion stage of the vesicle’s entrapped drug through its surface. Nanofibers, on the other hand, demonstrated only diffusion-related drug release through the core–shell structure of the nanofibers. Conventional anti-cancer drugs have low specificity since they destroy both cancerous and normal cells. To counteract this disadvantage, drug localization should be maintained following surgery. This could be achieved by using nanofibers, which provide sustained drug release at localized sites, lowering the risk of cancer recurrence after surgery [156]. It could potentially be placed directly at the tumor site for treatment purposes [157]. In turn, when nanoparticles are injected into veins, they are captured by the spleen and liver, limiting the drug’s specificity and efficiency [158]. Table 7 summarizes the factors that influence drug loading capacity, release profile, target specificity stability, and biocompatibility in nanofibers versus other nanocarrier systems.

### 7.1. Light-Responsive Smart Nanofibers with Embedded Nanoparticles

Jaswal et al. [164] synthesized polydopamine-coated gold nanospheres (GNSs@PDA) embedded in PCL electrospun nanofibrous composite scaffolds (termed PCL-GNSs@PDA) that were applied for photothermal bone cancer therapy and robust bone tissue regeneration. The prepared PCL-GNSs@PDA electrospun nanofibrous composite scaffolds were used as the NIR stimulus-responsive photothermal agent for bone cancer (MG-63) phototherapy as well as highly efficient osteopromotive scaffolds for bone tissue regeneration. PCL-GNSs@PDA (5.0 mg) nanofibrous scaffold was found to be the most efficient nanocomposite, resulting in 94% bone cancer cell ablation at 0.5 W/cm^2^ of NIR light power density as indicated by maximum cell alteration and damaged cytoskeleton as demonstrated by live/dead analysis. Fluorescence-activated cell sorting (FACS) analysis also revealed a high percentage of apoptotic cells (60.2%) compared to pure PCL (7.5%), suggesting that PCL-GNSs@PDA (5.0 mg) induced apoptosis in MG-63 cells. The presence of PDA-coated GNSs accelerated photo-hyperthermia as soon as NIR light was induced, resulting in increased cancer cell destruction due to the high electron density of the GNSs and their highly efficient light-to-heat conversion properties. To conclude, the synthesized PCL-GNSs@PDA electrospun nanofibrous composite scaffolds could be a potentially novel photothermal agent for bone cancer as well as a great candidate for bone tissue regeneration.

### 7.2. Light-Responsive Smart Nanofibers with Embedded Liposomes

Lem et al. [165] developed liposomes embedded in anionic nanofibrillar cellulose (ANFC) with tetracationic zinc phthalocyanine (ZnPc(MePyr)_4_) as a PhS in a hydrogel base outside of the liposomes. The ZnPc(MePyr)_4_ Phs and liposomes adhered strongly to the AFNC after simple mixing, making the system straightforward to build. When activated by light, ZnPc(MePyr)_4_ generated ROS, which disrupted the liposomal bilayer and, eventually, released the cargo. The release mechanism involved the generation of ROS and the subsequent oxidation of unsaturated lipids in the liposomal membrane. Following 5 min of light irradiation at 730 nm, 70% of calcein, a hydrophilic cargo molecule, was released at a low light dose (262 J/cm^2^). The developed system was exceptionally responsive to far-red light (730 nm, 0.875 W/cm^2^), allowing for deep tissue penetration. This novel cellulose-immobilized PhS liposomal complex could be used as a controlled drug delivery system, with potential application in cancer therapy.

### 7.3. Light-Responsive Smart Nanofibers with Embedded Hydrogels

Among the existing 2D nanomaterials, the burgeoning family of transition metal carbides, nitrides, or carbonitrides (MXenes) stands out for its unique combination of metallic conductivity, solution processability, high aspect ratio, and highly tunable properties [166]. MXenes have the chemical formula of M_n+1_X_n_T_x_ (n = 1–4), where M and X are early transition metals (e.g., Ti, V, Nb, Mo, etc.) and carbon and/or nitrogen, respectively, and Tx denotes distinct populations of surface-terminated groups (e.g., OH, O, and/or F). MXenes are of special interest in hydrogels due to their great mechanical strength [167], exceptional hydrophilicity [168], and rich surface chemistry, which offers another level of versatility [169]. Thus, when MXenes are incorporated into hydrogels, they provide improved features and new functions, resulting in increased performance. The distinct features of the resulting MXene-based gels are due to either the inherent properties of the MXenes, a combination of the functions of both MXenes and other components in the gel matrix, or even synergistic interactions between them. Indeed, the incorporation of MXenes into hydrogels not only enables the development of MXene-based soft materials with customizable features, but it also greatly improves MXene stability, which is frequently a limiting factor in many of its applications. MXene hydrogels have shown great potential in a variety of in vivo biological applications, including cancer therapy and drug release. MXene hydrogels provide a number of advantages over traditional hydrogels based on other 2D nanomaterials such as graphene, transition-metal dichalcogenides, and black phosphorus: (1) high hydrophilicity improves the dispersion and stability of MXene-derived photodynamic and photothermal agents in physiological fluids; (2) anticancer drugs can be easily grafted onto MXene surfaces with polar terminal groups; and (3) tuning the swelling performance of MXene hydrogels can result in excellent anticancer drugs with high loading capacities and high release rates [170]. For example, light-responsive MXene@Hydrogel composite nanofibers were developed by Yan et al. [171] using electrospraying and electrospinning techniques. Initially, they developed an electrospray heating device to maintain the phase transition temperature of the hydrogel. In contrast to conventional soaking methods, this device allowed the spraying of MXene@Hydrogel during the electrospinning process, resulting in a homogeneous distribution of the hydrogel. Because of the photothermal properties of the composite nanofibers, light was able to regulate the rheological characteristics of the hydrogel and hence provide drug release rate control. The introduction of Mxene into the hydrogel increased the mechanical strength of the hydrogel by increasing hydrogen bonding. As Mxene has a high thermal conductivity, a heating electrospray device was initially developed to increase hydrogel fluidity and adhere crosslinked hydrogel to the nanofiber. DOX was loaded into nanofibers and tested on glioma cells to ensure drug release control. In the absence of light, nanofibers had no discernible anticancer activity. After exposure to light, nanofibers demonstrated remarkable anti-cancer properties. These significant anticancer properties were primarily due to the photothermal effect of MXene, which was able to kill cancer cells, and the hydrogel reaching the phase transition point and releasing DOX, which also contributed to killing cancer cells. Another promising example is Wei et al. [172], who developed a polypyrrole (PPy)-coated copoly (isopropylacrylamide-4-benzoylphenyl acrylate) (P(NIPAM-ABP)) electrospun light-responsive hydrogel. The PPy-coated P(NIPAM-ABP) hydrogel was obtained via in situ polymerization of pyrroles on the nanofiber-oriented electrospun P(NIPAM-ABP) hydrogel. Compared to the original P(NIPAM-ABP) hydrogel, this PPy-coated P(NIPAM-ABP) hydrogel demonstrated significantly higher mechanical strength (5.12 MPa of tensile strength) and high-efficiency photothermal conversion over a wide range of wavelengths from 400 to 1600 nm. Because of their large specific surface area, the porous structure of the PPy-P(NIPAM-ABP) hydrogel nanofibers further increased the rate of light-responsive actuation. Consequently, an anisotropic robust bi-hydrogel (PCPP bi-hydrogel) actuator with powerful and ultrafast light-responsive deformation would be achieved by bonding a polyethylene glycol diacrylate-cellulose nanofiber (PEGDA-CNF) composite hydrogel membrane onto the PPy-P(NIPAM-ABP) hydrogel layer via interfacial UV-polymerization of PEGDA monomers. The anisotropic structure of P(NIPAM-ABP) nanofibers, on the other hand, allowed the PCPP bi-hydrogel actuator to give a variety of programmed, complicated deformations.

### 7.4. Dual Stimuli-Responsive Smart Nanofibers

In recent decades, researchers have attempted to improve tumor specificity and antitumor efficacy by combining two or more stimuli into a single vehicle, resulting in the development of dual-targeted nano-in-nanocarrier systems, such as nano-in-nanofiber emerging drug delivery systems. Dual-targeted stimuli-responsive smart nanofibers have several advantages over traditional nanofibers, such as targeting two or more receptors, improving tumor cellular internalization, releasing encapsulated drugs more efficiently and in a spatiotemporally controlled manner, and avoiding off-target toxicity to normal tissues.

#### 7.4.1. pH and Light-Responsive Smart Nanofibers

**Figure 10:**
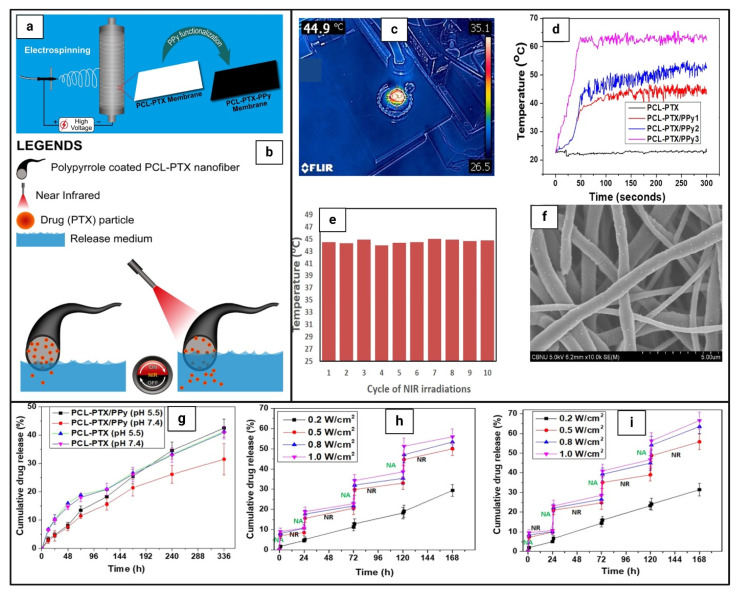
(**a**) A schematic representation of PPy-functionalized PCL-PTX membrane fabrication; (**b**) schematic illustration of the NIR-triggered drug release from the PPy-coated fiber; (**c**) NIR thermographic image of PPy-coated PCL-PTX mat (PCL-PTX/PPy1) under irradiation; (**d**) heating curves of different PPy-coated PCL-PTX mats under NIR laser power of 0.5 W/cm^2^; (**e**) stability assay; elevated temperature profiles of PPy-coated PCL-PTX mat (PCL-PTX/PPy1) over 10 cycles of exposure (laser on time: 5 min; laser off time: 30 min); (**f**) FE-SEM image of PPy-coated PCLPTX mat (PCL-PTX/PPy1) after 10 cycles of laser on/off process. NIR laser (808 nm) of power density of 0.5 W/cm^2^ was used for all experiments; (**g**) PTX-release profile of PCL-PTX and PCL-PTX/PPy membranes in PBS solutions with different pH values; (**h**,**i**) PTX-release profiles of PCL-PTX/PPy membranes with NIR irradiation in PBS solutions with pHs 7.4 and 5.5, respectively. Reprinted with permission from reference [173]. Copyright 2024 American Chemical Society.

Tiwari et al. [173] developed a pH- and NIR-responsive polypyrrole (PPy)-functionalized fibrous site-specific drug delivery platform with the goal of treating cancer by combining chemotherapy and photothermal ablation (Figure 10). First, a PTX-loaded PCL mat was prepared by electrospinning and was then surface-functionalized with various PPy concentrations. The resulting PPy-functionalized mats demonstrated excellent photostability and heating properties in response to NIR exposure. PPy-coated mats released more PTX at pH 5.5 than at pH 7.4. PTX release increased in response to NIR under both pH environments; however, pH 5.5 produced more release than pH 7.4, indicating a dual stimuli-responsive drug delivery platform that responded to both pH and NIR. More notably, 808 nm NIR irradiation significantly accelerated PTX release from PPy-coated PCL-PTX mats; this was followed by sustained release after laser irradiation was switched off, demonstrating representative progressive drug-release characteristics. PPy-coated PCL-PTX mats demonstrated significantly increased anticancer efficacy in vitro and in vivo when compared to PPy-coated PCL-PTX mats that were not exposed to NIR or uncoated mats (PCL-PTX). Cell viability was reduced to 10% for irradiated cancer cell types (CT26 and MCF7) at a laser power greater than 0.2 W/cm^2^, as measured by the CCK-8 assay for PPy-coated PCL-PTX membranes. Furthermore, irradiated cells at 0.2 W/cm^2^ or non-irradiated cells on PPy-coated PCL-PTX exhibited significantly higher cell viability than irradiated cells at higher laser powers. PPy-coated PCL-PTX fibers that were not exposed to NIR showed higher cell viability (87–89%) compared to the PCL-PTX fiber (80%). PPy coating enhanced the biocompatibility of fibers. Furthermore, the PPy coating on the fiber surface reduced the initial release of the encapsulated drug, indicating low toxicity. However, at 0.5 W/cm^2^, cell viability was reduced to 33 and 32% for CT26 and MCF7, respectively, which is significantly lower than the PCL-PTX mat, demonstrating that NIR irradiation is quite effective for cell death even without the drug. Under NIR irradiation, PCL/PPy showed significantly greater cell viability than the PCL-PTX/PPy mat. These findings are clearly related to the synergistic effect of NIR-triggered hyperthermia and drug release. Following the remarkable therapeutic outcome in vitro, the authors performed in vivo photothermal treatment in a xenograft mouse model using PPy-coated membranes. PCL-PTX and PCL-PTX/PPy mats (without NIR) increased tumor sizes in a time-dependent manner, reaching around 5 times the initial tumor sizes at the end of the study period, which were relatively smaller than those of the control samples. In contrast, mice treated with PCL-PTX/PPy with NIR radiation had tumor sizes reduced rapidly in an NIR-density-dependent manner. At the end of the treatment, average tumor sizes were reduced by 3 and 5 times for the PCL-PTX/PPy mats treated with 0.5 and 1 W/cm^2^ NIR light, respectively. PCL-PTX/PPy mats with NIR-treated mice groups showed substantial differences in tumor volume reduction compared to NIR-untreated groups. Mice treated with PCL-PTX/PPy with NIR light reduced tumor sizes shortly following the initial treatment period owing to rapidly induced hyperthermia and NIR-activated PTX release. Meanwhile, PCL-PTX/PPy (without NIR) failed to suppress tumor growth, possibly due to the delayed release of PTX in the tumor environment in the absence of NIR. These findings suggested that the excellent NIR-responsiveness of PPy played an important role in tumor reduction. To conclude, NIR-treated PPy-coated meshes exhibited increased cell death by a combination of NIR-triggered hyperthermia and an activatable drug-release mechanism. The developed system would serve as a multifunctional platform for cancer treatment. 

**Figure 11:**
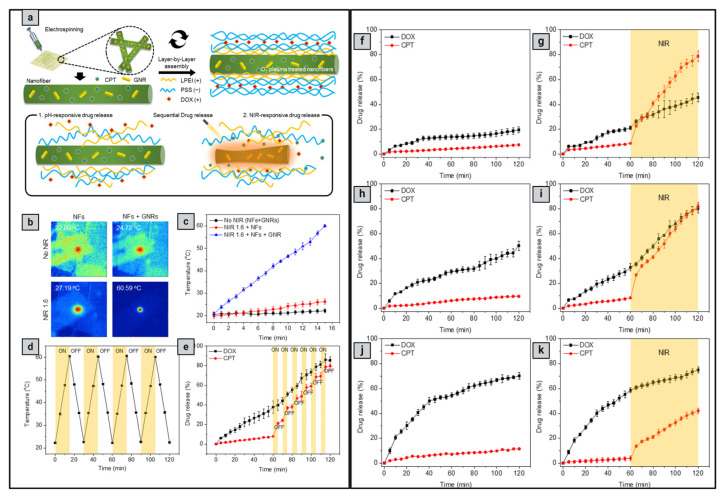
(**a**) Light-responsive LbL assembled nanofibers for sequential drug release; (**b**) IR camera images of temperature variation of nanofibers with and without NIR exposure in the absence and presence of gold nanorods; (**c**) temperature change due to NIR radiation on nanofiber in water; (**d**) rate of temperature increase of nanofibers due to cyclic on-off NIR exposure; (**e**) drug release through NIR exposure in cyclic on-off pattern; (**f**,**g**) sequential drug release at pH 7.4; (**h**,**i**) sequential drug release at pH 6; (**j**,**k**) the rate determination of sequential and on-demand drug release at low pH and NIR. Reprinted with permission from reference [142]. Copyright 2024 Elsevier.

Recently, Singh et al. [142] developed layer-by-layer (LbL)-assembled smart nanofibers for multi-stimuli-triggered sequential drug release in skin cancer treatment (Figure 11). CPT, an anticancer drug, was encapsulated in nanofibers using electrospinning, and positively charged DOX was combined with an anionic electrolyte via LbL assembly. The resulting LbL-nanofiber platform exhibited excellent loading efficiency, stability, and a very high surface-area-to-volume ratio, allowing for effective and sequential drug release. DOX was released more rapidly at pH 6, but less effectively at pH 7.4, while CPT was released under regulated conditions after being exposed to NIR radiation. Because the PNIPAM polymer, which controls CPT release, is thermally sensitive, the increased temperature caused by gold nanorods under NIR illumination can alter nanofiber expansion and contraction. Cell studies confirmed the efficacy and biocompatibility of nanofibers. DOX and CPT inhibited B16-F10 cell proliferation through sequential and on-demand release, depending on pH, NIR exposure time, and therapeutic dose. A cell viability study with B16-F10 melanoma cells was carried out to evaluate the treatment efficacy of this approach using the MTT assay. Cell toxicity was assessed following NIR irradiation without nanofibers or drug treatment. However, when the intensity of NIR light increased, cell viability decreased slightly. After 48 h, free CPT and DOX demonstrated 9.4 ± 1.5% and 21.4 ± 1.8% cell viability at 5 μg/mL, respectively. Cell viability was measured at all illumination times after 24 and 48 h of incubation. Cell death increased by 82.9 ± 3.9%, 74.4 ± 3.4%, and 58.2 ± 4.4% when LbL-NF was submerged for 1, 5, and 10 min, respectively, after 24 h of incubation. DOX was also released. However, after 48 h of incubation, cell viability was 56.7 ± 4.2%, 43.2 ± 2.6%, and 31.9 ± 3.8% for dipping periods of 1, 5, and 10 min. In contrast, releasing CPT after 1, 5, and 10 min of NIR illumination improved the anticancer effects of LbL-nanofiber. After 24 h of CPT and DOX release, cell viability reached 51.3 ± 1.4%, 42.0 ± 5.3%, and 32.4 ± 1.4% for 1, 5, and 10 min, respectively. After 48 h, cell viability was 33.1 ± 3.2%, 24.7 ± 3.4%, and 11.0 ± 1.9% after 1, 5, and 10 min, respectively. LbL-nanofiber released less drugs than free CPT and DOX. This approach offers great potential for treating a wide range of complex diseases, including wound healing and skin cancer.

#### 7.4.2. Thermo- and Light-Responsive Smart Nanofibers

**Figure 12:**
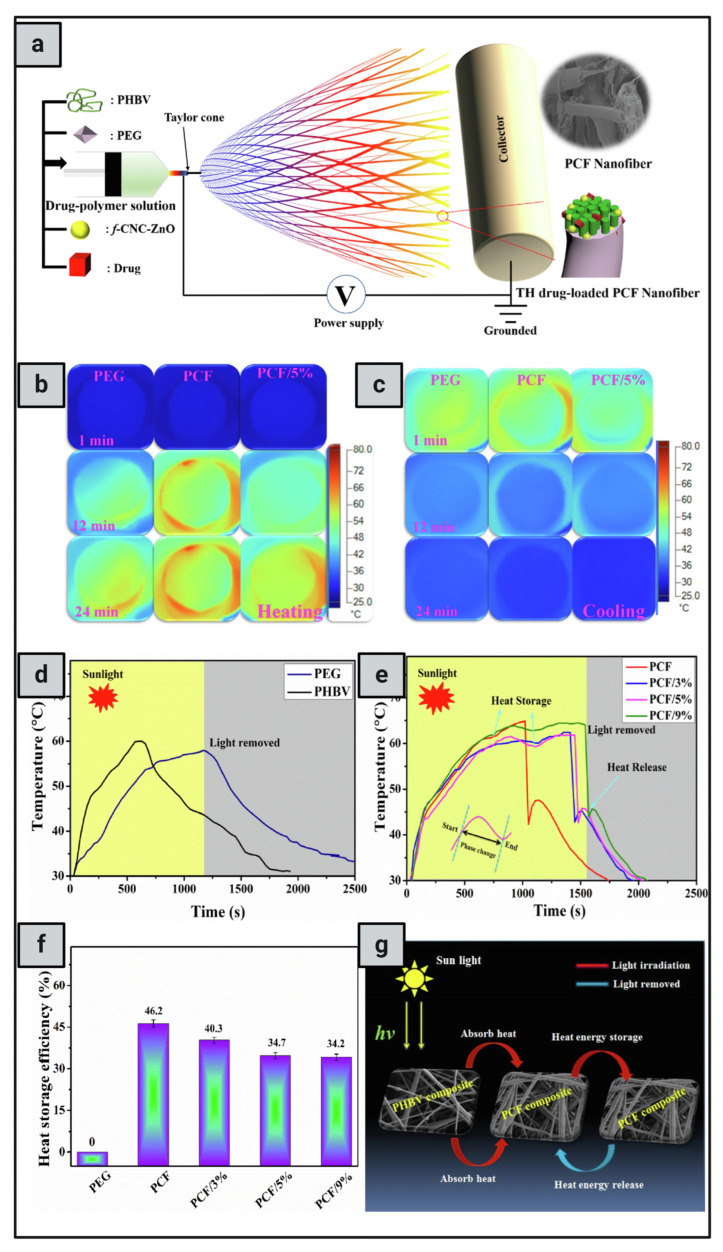
(**a**) Schematic illustrating possible electrospinning process for preparing drug-loaded PCF composite encapsulated with CNC-ZnO; thermal images of PEG (left), PCF, and PCF/5% composite (right) during (**b**) heating, and (**c**) cooling for 1, 12, and 24 min; heat conversion storage, and release curves for PEG (**d**) and PCF (**e**) composite with various contents of CNC-ZnO under solar illumination intensity at 100 mW cm^−2^; (**f**) heat storage efficiency; (**g**) schematic diagram of light to heat conversion and storage. Reprinted with permission from reference [174]. Copyright 2024 Elsevier.

Abdalkarim et al. [174] employed electrospinning to produce thermo- and light-responsive functionalized cellulose nanocrystal-zinc oxide (f-CNC-ZnO) nanohybrid-based poly(3-hydroxybutyrate-co-3-hydroxy valerate) (PHBV) phase transition nanofiber (PCF) composites with substantial thermal energy storage capacity (Figure 12). Under sunlight, PCF composites (without f-CNC-ZnO) absorbed light and stored heat energy with an efficiency of 46.3%. PCF composites revealed an “on–off” temperature regulation of tetracycline hydrochloride (TH) release based on phase transition temperatures. Because of the extremely slow diffusion through a solid matrix, very little TH was released at temperatures below the melting point. Beyond the melting point, the encapsulated TH was readily separated from the molten PCF composite. In simulated normal physiological conditions, the resulting TH drug-loaded PCF composites demonstrated rapid release, making them ideal candidates for controlled drug release systems. Thus, PCF composites with excellent thermo- and light-responsive properties, as well as high energy storage efficiency, are promising for long-term thermal storage and controlled drug delivery systems for cancer therapy.

#### 7.4.3. Dual- or Multi-Stimuli-Responsive Nano-in-Nanofiber for High-Precision Tumor Targeting

**Figure 13:**
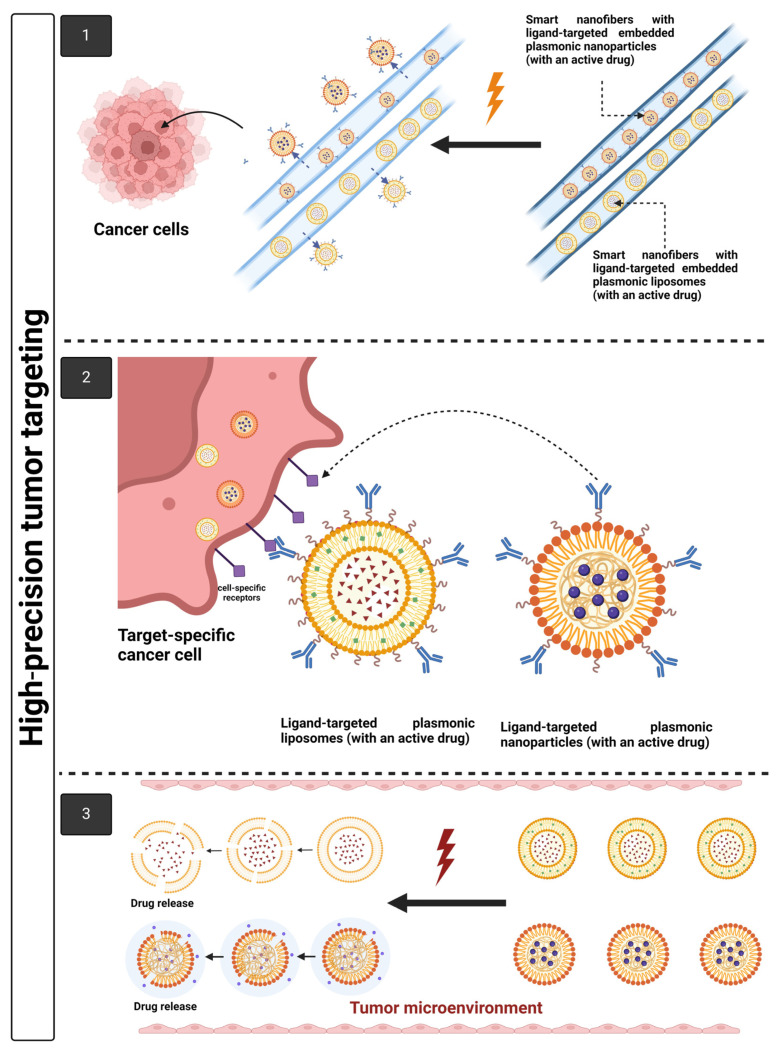
A schematic illustration of a hybrid dual-responsive system with nanoparticles or liposomes embedded in nanofibers for high-precision tumor targeting. (1) The release of plasmonic liposomes or nanoparticles from UV-sensitive nanofibers; (2) ligand-targeted plasmonic liposomes or nanoparticles recognize and bind to cell-specific receptors on target cells; (3) controlled release of an active drug from NIR-responsive plasmonic liposomes or nanoparticles into the tumor microenvironment and at a specific tumor site. Created with BioRender.com.

Combining two or more stimuli into two emerging vehicles enables high-precision tumor targeting, culminating in the development of dual-targeted, dual-responsive nano-in-nanocarrier systems, such as dual-responsive nanoparticle-in-nanofiber or liposome-in-nanofiber emerging drug delivery systems, in which nanofibers can respond to one stimulus and nanoparticles or liposomes can respond to another or the same stimulus at different conditions. To the best of the authors’ knowledge, these hybrid dual-responsive systems have not been investigated thoroughly, but they could pave the way for precision cancer treatment. Figure 13 shows the development of UV-responsive nanofibers carrying NIR-responsive nanoparticles or liposomes with active drugs.

## 8. Challenges and Future Prospects

In recent years, there has been increasing emphasis on integrating sustainable and eco-friendly design concepts into nanomaterials. However, electrospun and forcespun nanofibers usually fail to meet environmental sustainability criteria due to their dependency on solvents, which have substantial drawbacks such as toxicity, flammability, and disposal difficulties. Due to limited solvent options and increased regulatory and safety concerns, it has become essential to find alternative eco-friendly solvents. The choice of solvent is critical in electro- and forcespinning techniques since it dissolves and evaporates the polymer during the spinning process. However, many commonly used organic solvents are not only toxic but also harmful to the environment [175,176]. These solvent residues hinder the scalability and diversity of applications for electro- and forcespun nanofibers, particularly in the biomedical field. To overcome these challenges and promote a more eco-friendly and sustainable approach, traditional electrospinning and forcespinning techniques are required to be upgraded. Green electrospinning involves the use of raw materials that are biobased, biodegradable, and eco-friendly. The solutions used in green electrospinning are non-toxic and environmentally safe, and the technique is entirely solvent-free, making it both efficient and sustainable. Green electrospinning can be classified into different categories based on the materials, solvents, and techniques used. These include green solution electrospinning [177], solvent-free electrospinning [175], colloid electrospinning [178], and green and degradable material electrospinning. Although green electrospinning has several advantages, its use is still limited by the following challenges: (1) fibers have a large diameter due to the poor conductivity and high viscosity of the solvent; (2) the equipment is relatively complicated and requires additional modifications (e.g., melt and thermo-curing electrospinning require a heater; UV-curing electrospinning requires a nitrogen chamber); (3) green electrospinning may produce nanofibers with slightly lower mechanical performance than traditional electrospinning methods that use stronger but toxic solvents. This may limit their use in certain applications; (4) scaling up green electrospinning processes can be challenging owing to the necessity for precise control of environmental conditions like humidity and temperature; (5) some polymers may not be easily soluble in green solvents, limiting the variety of materials that can be employed; and (6) not all green materials are as readily accessible as conventional polymers and solvents. This can result in increased material costs or limited availability [179].

Despite significant recent advances in fiber-forming spinning processes, implementing electrospinning and forcespinning with stimuli poses a major obstacle to their development and application. Therefore, more research-oriented nanofiber-stimuli responsiveness is required in order to advance towards the next generation of dual-controlled, stimuli-responsive nanofibers. Light, an external stimulus, is one of the most promising triggers in nanomedicine to activate on-demand drug release from nanofibrous systems. Light-triggered drug release can be achieved through light irradiation at various wavelengths, such as UV, visible, or even the NIR region, depending on the light source used, the photophysical properties of the photoresponsive material embedded in the nanofiber matrix, and the structural characteristics and material composition of the nanofibrous system. A variety of light sources have been developed for potential cancer diagnostic and therapeutic applications. These light sources include lasers (250–900 nm), light-emitting diodes (LEDs, 250–700 nm), mercury arc lamps (250–600 nm), and X-rays. Lasers are widely used to treat cancer cells due to their superior monochromatic performance and ability to centralize into a coherent beam with extremely high energy density in a very short period of time, which can be a few microseconds or even milliseconds. Z-Bolt^®^ Electro-Optics provides a collection of high-powered, constant-on laser pointers suitable for benchtop experimentation and small-area irradiation. Though highly focused, laser pointers cannot easily change intensity, so experiments testing variable light intensities are challenging [180,181]. LEDs are highly directional light sources for use in cancer diagnosis and therapy. Various manufacturers offer a wide range of wavelengths and intensities. ThorLabs, Inc. provides multiwavelength LED arrays for microscopy that can be easily adapted for light delivery on the benchtop. Individual LEDs can also be purchased from companies (such as LED Engin, Inc.) to allow users to control wavelength and power. Compared to lasers, LEDs have several advantages and unique photophysical properties, such as: (1) they are relatively easy and safe to handle because they do not require a high voltage; (2) they can be integrated into digital systems, allowing for complex light-setting programs such as varying spectral composition over the course of phototherapy or during different treatment stages [182,183]. Excelitas Technologies Corp. offers broad-spectrum mercury-arc lamps that can be adjusted to specific wavelengths using bandpass filters. These lamps typically capture large outputs at 254, 365, 405, 436, 546, and/or 579 nm. These light sources are quite affordable and powerful. X-rays, another type of ionizing radiation, are frequently employed in radiotherapy. The high energy of X-ray radiation causes DNA damage, either through ionization or the formation of cytotoxic free radicals, resulting in apoptosis [184].

The application of light-responsive smart nanofibers for medical use is currently very limited by light penetration depth; however, recent technological advances in fiber optics, such as fiber optic endoscopy (FOE), have allowed for the temporary placement of optics to target deep tissues [185]. The future of endoscopy will be dictated by rapid technological advancements in the development of light sources, optical fibers, and miniature scanners that will enable images to be acquired across multiple spectral regimes, with deeper tissue penetration, and in three-dimensional form. Furthermore, novel strategies in photoresponsiveness, such as the development of photocleavable groups that can be activated by long wavelengths [186] or by efficient up-conversion systems [187], have resulted in increased tissue penetration depth. On the other hand, the relationship between light-stimulus-responsive phase behavior and nanofibrous surface structure is still a subject of debate in order to investigate the rational design of composite nanofibers with multifunctional properties and excellent stimulus-responsiveness to light. This could be achieved by selecting the proper polymers, co-polymers, solvents, and co-solvents, optimizing spinning process parameters, selecting the proper geometrical architecture of nanofibers (monolithic, core–shell, or layer-by-layer), the photophysical properties of the photoresponsive material embedded in the nanofibrous system, and the degree of surface functionalization.

Several in vitro proof-of-concept studies on light-stimulus-responsive smart nanofibers have been published; however, none have reached the clinic. The toxicity of these smart, responsive drug delivery systems is unclear and is determined by a variety of factors, including material composition, physicochemical and photophysical properties of the photoresponsive materials, dosage form, and route of administration. Their composition, drug release kinetics, and transport mechanisms are more complex than conventional drug delivery systems, making it more difficult to assess their side effects and toxicities. Furthermore, the clinical usefulness of these smart, responsive nanofibrous systems is limited due to the complexity of their architectures and designs. In addition, several safety and regulatory challenges must be overcome first before nanofibers can be successfully translated into the clinic, particularly the strict requirements of the FDA and European Medicines Agency (EMA) that ensure the safety and efficacy of these systems. In respect to industrial applicability, the application of fiber-forming spinning processes for industrial use is limited to small-scale and proof-of-concept studies due to the high cost of materials, manufacturing processes, optimization, and validation. Therefore, it is essential to develop cost-efficient and scalable fiber-forming spinning processes with high-throughput fiber production rates and stimulus responsiveness.

The combined use of spinning and 3D printing technologies [188,189,190] is predicted to grow in the next few years, allowing for the development of smart, responsive, multifunctional nanoplatforms with novel advanced characteristics and tailored designs and functionalities. Furthermore, the application of artificial intelligence (AI) [191,192,193] could be used in smart nanofibers to propose nanofiber-drug combinations based on biopharmaceutical drug classification, drug–polymer interactions, polymer dissolution, and compatible solvent systems. Additionally, AI could be applied to remodel the tumor microenvironment to overcome drug resistance and enhance drug delivery and efficacy. Examples of these AI systems are artificial neural networks (ANN), memetic algorithm optimization, genetic function approximation (GFA), and adaptive neuro-fuzzy inference systems (ANFIS). Among these systems, ANN is the favored modeling methodology and the most effective technology for developing new techniques. It consists of several layers of interconnected nodes that can process and learn from data. ANN can improve the effectiveness of nanofibers, change their structure, and alter their delivery and release mechanisms. This is feasible because of the unique design of ANN, which allows it to simulate the behavior of neurons in the human brain, which communicate by electrical and chemical impulses [194,195].

## 9. Conclusions

Despite the fact that there are several cancer treatment options available, such as chemotherapy, hormone therapy, immunotherapy, PDT, PTT, and surgery, the ideal treatment for cancer is still being investigated. Significant progress has been made in this direction, thanks to developments in nanotechnology. Nowadays, many nano-delivery systems have been developed for the treatment of cancer, and they have proven to be safe chemotherapeutic options owing to their selectivity, specificity, and targetability, as well as their high internalization into tumor cells. Electrospun and forcespun nanofibers—which offer high porosity, high surface area-to-volume ratios, high tunability, controlled drug release properties, high encapsulation efficiency and drug loading capacity, biodegradability, biocompatibility, and affordability—are excellent examples of these systems with exceptional physicochemical and mechanical properties. Furthermore, because they can be customized to respond to specific stimuli, such as light, they are excellent candidates for controlled, on-demand, targeted drug delivery and release. Moreover, nanoparticles, liposomes, hydrogels, and smart nanofibers can be combined to form a hybrid system with controlled release. Several studies have demonstrated the concepts of smart nanofibers for controlled release; nevertheless, converting these smart nanofibers to clinical applications may be time-consuming. Even with all of these promising characteristics, nanofibers still face a number of challenges that need to be overcome before they can be used in therapeutic and clinical settings. Future research should focus on commercialization and bringing innovative technologies for smart nanofiber design to the market to help bridge the gap between lab-to-market research. Furthermore, future research should focus on developing smart nanofibers that respond to various stimuli, especially light, under normal physiological conditions. Light remains unique compared to other stimuli. It has several advantages over other stimuli, including providing a wide range of effective and relatively safe stimulus levels; triggering various molecular processes orthogonally and sequentially; and being localized to specific sites within human tissues and organs to elicit a particular biological response, i.e., spatiotemporal control over the therapeutic window to minimize off-target activity and maximize therapeutic efficacy.

## Figures and Tables

**Table 1:** The parameters and factors that influence electrospun nanofiber production.

Parameter	Factor	Impact	Reference
**Polymer solution parameters**	- Higher polymer molecular weight	- Higher uniformity of nanofibers	[9]
	- Increased polymer concentration	- Larger nanofiber diameter	[9]
	- Increased polymer solution viscosity	- Larger nanofiber diameter	[10]
	- Increased polymer conductivity	- Smaller nanofiber diameter	[9]
**Processing parameters**	- Elevated humidity	- Higher porous structures	[18]
	- Increased voltage difference	- Smaller nanofiber diameter	[19]
	- Increased the feed solution flow rate	- Larger nanofiber diameter	[9]
	- Increased distance between the solution surface and the collector	- Smaller nanofiber diameter	[14]
	- Type of collector	- By changing the collector type (flat plate, drum, mandrel, etc.), different fiber diameter, morphology, structure, and alignment can be obtained.	[12,13]

**Table 2:** The parameters and conditions that influence drug release from electrospun nanofibers.

Parameter	Factor	Impact	Reference
**Drug-related parameters**	- Drug solubility	- Hydrophobic drug release is more controllable than hydrophilic release. - The more soluble the drug is in the polymer solution, the better it disperses inside the nanofiber matrix, resulting in uniform release.	[21,22]
	- Drug loading	- Increased drug loading results in a less controlled release.	[10]
**Polymer-related parameters**	- Crosslinking	- Polymer crosslinking is used to produce a more controlled release.	[11]
	- Addition of inorganic nanocarrier	- The drug will be adsorbed on the surface of inorganic nanoparticles before being dispersed in the polymer solution, overcoming the barrier of low drug-polymer interaction.	[23]
	- Ratio of amorphous to crystalline composition	- Modify the drug release mechanism to rely solely on diffusion or on diffusion and matrix degradation.	[24]
	- Polymer hydrophilicity	- Hydrophilic polymers are preferred over hydrophobic polymers for immediate release.	[25]
**Process parameters**	- Monoaxial or uniaxial electrospinning	- Encompass a single capillary nozzle for the development of smooth and continuous nanofibers. - It can be set up to spin either horizontally or vertically. - Produce nanofibers with burst or sustained release profiles.	[26]
	- Coaxial electrospinning or co-electrospinning	- Encompass a two-needle spinneret for the development of core–shell nanofibres, in which one needle is concentrically inserted inside the other. - Produce core–shell structures with controlled release profiles. - Provide excellent flexibility in the selection of materials and drugs.	[26]
	- Triaxial electrospinning	- Encompass a spinneret with three concentric needles for the development of multistage structured nanofibers. - Produce a three-layer microstructure composed of core, intermediate, and sheath region. - Provide a combined delivery of multiple drugs with different release patterns.	[26]

**Table 3:** Polymers used for the fabrication of electrospun nanofibers.

Polymer Name	Functional Properties	Reference
- **Poly-ε-caprolactone (PCL)**	- PCL has unique mechanical properties, miscibility, potency, and biodegradability.	[28,29]
- **Co-doped calcium titanate (CaTiO_3_): erbium and ytterbium co-doped fibers functionalized with poly(acrylic acid)**	- Near-infrared (NIR)-triggered and optically monitored drug release.	[30,31]
- **Poly(N-isopropylacrylamide) (PNIPAM) with gold nanorods**	- When gold nanorods are exposed to NIR radiation, the heat generated changes the size of the PNIPAM nanofibers, allowing the encapsulated drugs to be released.	[32,33]
- **Melanin nanoparticle-PCL nanofiber (MNPs-PCL)**	- The MNPs-PCL hybrid system exhibited controlled and sustained drug release. - Malignant melanoma cells were photodynamically treated by activating the MNPs-PCL system with UV-A light.	[34]
- **Black phosphorus (BP) nanosheets, silk fibroin (SF), and polylactic-co-glycolic acid (PLGA)**	- The hybrid system demonstrated excellent photothermal properties, which were controlled by changing the BP@SF content and the intensity of NIR light. - In vitro, it was found to be effective at killing HepG2 cancer cells.	[35]

**Table 4:** The parameters and factors that influence forcespun nanofiber production.

Parameter	Factor	Impact	Reference
**Polymer solution or melt parameters**	- Concentration	- Bead formation typically arises at low polymer concentrations. - Increasing the polymer concentration leads to a mixture of beads and fibers. - Beyond a critical concentration point, continuous fiber development ensues, facilitated by sufficient chain entanglement.	[45,46,47]
	- Molecular weight	- Increasing molecular weight leads to a rise in inherent viscosity. - Upon reaching a threshold viscosity, fibers transition from a round to a flat shape. - This transition involves an increase in both fiber diameter and inter-fiber spacing.	[48,49]
	- Viscosity	- Higher polymer network entanglement correlates with increased solution viscosity. - Elevated viscosity restricts solvent evaporation, preventing jet splitting, stretching, and thinning. - Highly viscous solutions and extended stress relaxation periods promote the formation of larger fiber diameters.	[46,47]
	- Surface tension	- Centrifugal force must exceed surface tension force. - Bead formation is influenced by altering the surface tension of the polymer solution. It occurs due to reduced surface tension in spherical geometries, a factor that can be mitigated by adjusting polymer concentration.	[50,51,52]
**Processsing parameters**	- Rotational speed and flow rate	- Higher rotational speeds are required for defect-free fibers with less volatile, aqueous solvents, while lower spinning speeds are suitable for highly volatile solvents to achieve continuous fiber formation. - Bead formation shows an inverse relationship with rotational speed. - A slow flow rate extends solvent evaporation during spinning, allowing wet fibers to accumulate on collectors and form thin films.	[52,53,54,55]
	- Nozzle-collector distance	- Closer needle–collector spacing leads to porous fiber, while spacing beyond a critical distance produces regular, dense fiber. - If the nozzle-to-orifice spacing is too large, the rotating jet fails to reach the collectors.	[54,56]
	- Air foil	- A longer air foil improves fiber production by aiding capillary thinning of the liquid jet before extensive solvent evaporation.	[57]
	- Ambient parameters	- Temperature significantly impacts fiber characteristics. - Elevated humidity levels hinder fiber generation, resulting in thin film formation on the collector.	[47,58]
	- Types of collectors	- Column collectors - Vacuum collection system - Deep dish collector - Polypropylene substrate with an air-free box (produce non-woven fibers)	[59,60]
	- Nozzle geometry	- Nozzle length can impact jet continuity and stabilized flow. - Smaller nozzle diameters are needed for nanofiber production. - Decreasing the orifice length-to-diameter ratio results in larger-diameter fibers.	[47,54]

**Table 5:** Polymers used for the fabrication of forcespun nanofibers.

Polymer Name	Functional Properties	Reference
**TAF**	- TAF mats exhibited superhydrophobic properties, with contact angles up to 169° ± 3°. - TAF mats demonstrated excellent mechanical properties, enduring 17.5 MPa of mechanical stress and a Young’s modulus of 348 MPa, as well as exceptional thermal stability.	[47]
**PAN**	- Sodium chloride (NaCl) addition decreased carbon fiber diameter by 37% and improved graphitization in PAN solution. - X-ray photoelectron spectroscopy (XPS) analysis showed increased surface functionality in PAN/NaCl precursor fibers, leading to enhanced electrochemical performance compared to pure PAN fibers.	[75]
**PVP**	- High-speed rotary spinning produces micro- or nanofibers of PVP and PVP-VA with optimal morphologies. - Positron lifetime distributions revealed changes in fiber-free volumes based on solution composition. - Modifying polymer type and alcohol–water ratios altered fiber supramolecular structure, influencing morphological and mechanical characteristics for solid dosage form processing.	[76]
**SF**	- Centrifugal spinning transformed the conformations of the SF from random coil to β-sheet while also enhancing crystallinity, orientation, and thermostability.	[58]
**α-Fe_2_O_3_**	- Centrifugal spinning with an iron precursor/polymer aqueous solution was employed. - The fibers exhibited an average wall thickness of 55 ± 15 nm and an outer diameter of 852 ± 86 nm.	[77]
**EC**	- The addition of 30% water to the binary solvent system increased the EC/PVP fiber-specific surface area significantly. - However, achieving a porous structure under the same condition was not possible, indicating the strong influence of the spinning method on jet diameter and solvent evaporation.	[78]
**PCL**	- Increasing rotational speed led to a decrease in fiber diameter. - At 9000 rpm, the average diameter was 220 ± 98 nm.	[60,63]
**PEO**	- Fiber size decreased as rpm increased, reaching an optimum range for high yields. - Using a less volatile solvent could result in smaller fiber diameters.	[79]
**Nylon-6**	- The results demonstrated successful deposition of evaporated silver and copper nanoparticles onto nylon-6 nanofibers, forming thin, adherent films while maintaining native fiber morphology and achieving electrically conductive nanostructures.	[80]
**PS**	- Centrifugal spinning demonstrated potential in mass-producing superhydrophobic micro- and nanofibers to meet rising demand.	[59]
**PLLA**	- Thermal analysis confirmed immiscibility and complete solvent evaporation. - In vitro testing showed good cytocompatibility.	[81,82]
**PAN**	- PAN fibrous mats offered a cost-effective method for mass-producing high-quality fibrous mats, heralding a revolution in large-scale fiber production.	[83]

**Table 6:** Different designs of smart nanofibers with photoresponsive properties.

Design	Drug	Photoresponsive Material or Nanocarrier	Polymer	Cancer Type	Reference
**Monolithic**	______	AuNRs	PEG	Lung, breast, and cervical cancer	[128]
	______	PDA	PCL	Colorectal cancer	[132]
	DOX	PLLA and MWCNTs	______	Cervical cancer	[133]
	______	GO/AuNRs	PCL	Breast cancer	[144]
	DOX	MoS2	Chitosan/PVA	Colorectal cancer	[126]
**Core–shell**	DOX	AuNCs	PCL	Breast cancer	[139]
	PTX	GO/AuNRs	PTMG-PU and chitosan	Lung cancer	[140]
	CAR	RB	Eudragit L100-55 and HPMC	Colon cancer	[141]
**Layer-by-layer assembly**	DOX and CPT	AuNRs	______	Skin cancer	[142]
	DOX	PDA	PCL	Lung and breast cancer	[143]

**Table 7:** Factors that influence drug loading capacity, release profile, target specificity stability, and biocompatibility in nanofibers versus other nanocarrier systems.

Nanocarrier System	Drug loading Capacity	Release Profile	Targeting Specificity	Stability	Biocompatibility	Reference
**Nanofibers**	- Drug and polymer used in nanofiber preparation. - Method of preparation and drug incorporation.	- Polymer used in nanofiber preparation. - Method of drug preparation and drug incorporation. - Surface functionalization. - Fiber diameter and pore size.	- Polymer used in nanofiber preparation. - Surface functionalization and coating.	- Drug and polymer used in nanofiber preparation. - Surface functionalization and coating.	- Polymer used in nanofiber preparation. - Surface functionalization and coating.	[9,12]
**PLGA nanoparticles**	- Drug used in nanoparticle preparation.	- Drug used in PLGA nanoparticle preparation. - Surface functionalization. - Size and particle distribution.	- Surface functionalization and coating.	- Drug used in nanoparticle preparation. - Surface functionalization and coating.	- Surface functionalization and coating.	[159,160]
**Solid lipid nanoparticles**	- Drug, lipid phase composition, and the type of surfactant. - Surface functionalization. - Method of emulsification and drug incorporation.	- Drug, lipid phase composition, and surfactant type. - Surface functionalization. - Method of emulsification and drug incorporation. - Size and particle distribution.	- Surfactant type. - Surface functionalization and coating.	- Drug, lipid phase composition, and surfactant type. - Surface functionalization and coating. - Method of emulsification and drug incorporation.	- Lipid phase composition and surfactant type. - Surface functionalization and coating.	[161,162]
**Liposomes**	- Drug and lipid phase composition. - Method of preparation and drug incorpotation. - Liposome structure and size.	- Drug and lipid phase composition. - Method of preparation and drug incorpotation. - Liposome structure and size.	- Lipid phase composition. - Surface functionalization and coating.	- Drug and lipid phase composition. - Method of preparation and drug incorpotation. - Surface functionalization and coating.	- Lipid phase composition. - Surface functionalization and coating.	[114,163]

## Data Availability

Not applicable.

## Abbreviations

List of abbreviations and acronyms used in this review.

**Abbreviation**	**Meaning**
**Ac-DEVDD-TPP**	Ac-Asp-Glu-Val-Asp-Asp-TPP
**AFM**	Atomic force microscopy
**ANFIS**	Adaptive neuro-fuzzy inference systems
**ANFC**	Anionic nanofibrillar cellulose
**ANN**	Artificial neural networks
**AuNCs**	Gold nanocages
**AuNRs**	Gold nanorods
**BP**	Black phosphorus
**CAR**	Carmofur
**Casp3**	Caspase-3
**CaTiO_3_**	Co-doped calcium titanate
**CLSM**	Confocal laser scanning microscopy
**CMCs**	Carboxymethyl chitosan
**CPT**	Camptothecin
**CDs**	Cyclodextrins
**DCC**	N,N′-dicyclohexylcarbodiimide
**DCM**	Dichloromethane
**DHT**	Dehydrothermal
**DMAP**	4-dimethylaminopyridine
**DMF**	Dimethylformamide
**DNA**	Deoxyribonucleic acid
**DOX**	Doxorubicin
**DSC**	Differential scanning calorimetry
**EC**	Ethyl cellulose
**ECM**	Extracellular matrix
**EMA**	European Medicines Agency
**FACS**	Fluorescence-activated cell sorting
**f-CNC-ZnO**	Functionalized cellulose nanocrystal-zinc oxide
**FDA**	U.S. Food and Drug Administration
**FOE**	Fiber optic endoscopy
**FT-IR**	Fourier-transform infrared spectrometry
**GFA**	Genetic function approximation
**GMA**	Glycidyl methacrylate
**HDF**	Human dermal fibroblast
**HFIP**	Hexafluoroisopropanol
**IG**	Indocyanine green
**LEDs**	Light-emitting diodes
**LbL**	Layer-by-layer
**MC**	Merocyanine
**MNPs**	Melanin nanoparticles
**MNPs-PCL**	Melanin nanoparticle-PCL nanofiber
**MWCNTs**	Multiwalled carbon nanotubes
**NaCl**	Sodium chloride
**NIR**	Near-infrared
**NMR**	Nuclear magnetic resonance
**NNHs**	Nanoparticle-nanofiber hybrids
**OSCC**	Oral squamous cell carcinoma
**PAB**	4-propargyloxyazobenzene
**PAN**	Polyacrylonitrile
**PCF**	Phase change nanofiber
**PCL**	Polycaprolactone
**PDA**	Polydopamine
**PDT**	Photodynamic therapy
**PEG**	Polyethylene glycol
**PEGDA-CNF**	Polyethylene glycol diacrylate-cellulose nanofiber
**PEO**	Polyethylene oxide
**PHBV**	Poly(3-hydroxybutyrate-co-3-hydroxy valerate)
**P(NIPAM-ABP**	Copoly (isopropylacrylamide-4-benzoylphenyl acrylate)
**PhS**	Photosensitizer
**PLA**	Polylactic acid
**PLGA**	Polylactic-co-glycolic acid
**PLLA**	Poly(L-lactic acid)
**PMPC-*b*-PCL**	Poly(2-methacryloyloxyethyl phosphorylcholine)-*b*-poly(ε-caprolactone)
**PNIPAM**	Poly(N-isopropylacrylamide)
**POSS**	Polyhedral oligomeric silsesquinoxanes
**PPy**	Polypyrrole
**PTMG-PU**	Poly(tetramethylene ether)glycol-based polyurethane
**PTT**	Photothermal therapy
**PTX**	Paclitaxel
**PU**	Polyurethane
**PVA**	Polyvinyl alcohol
**PVP**	Polyvinylpyrrolidone
**PVP-VA**	Polyvinylpyrrolidone and vinyl acetate
**RAFT**	Reversible addition fragmentation chain-transfer
**RB**	Rose Bengal
**RhB**	Rhodamine-B
**ROS**	Reactive oxygen species
**SEM**	Scanning electron microscopy
**SF**	Silk fibroin
**SP**	Spiropyran
**TAF**	Teflon^®^ AF
**TCPP**	5,10,15,20-tetrakis (4-carboxyphenyl) porphyrin
**TEM**	Transmission electron microscopy
**TFA**	Trifluoroacetic acid
**Tg**	Glass transition temperature
**TH**	Tetracycline hydrochloride
**THF**	Tetrahydrofuran
**UV**	Ultraviolet
**UVA**	Ultraviolet light A
**VBC**	Vinyl benzyl chloride
**XPS**	X-ray photoelectron spectroscopy
**XRD**	X-ray diffraction
**ZnPc(MePyr)4**	Zinc phthalocyanine
**5FU**	5-fluorouracil
**α-Fe2O3**	Iron sesquioxide, hematite
